# On the Relationships of Postcanine Tooth Size with Dietary Quality and Brain Volume in Primates: Implications for Hominin Evolution

**DOI:** 10.1155/2014/406507

**Published:** 2014-01-30

**Authors:** Juan Manuel Jiménez-Arenas, Juan Antonio Pérez-Claros, Juan Carlos Aledo, Paul Palmqvist

**Affiliations:** ^1^Departamento de Prehistoria y Arqueología, Facultad de Filosofía y Letras, Campus de Cartuja S/N, 18071 Granada, Spain; ^2^Edificio Centro de Documentación Científica, Instituto Universitario de la Paz y los Conflictos, Universidad de Granada, C/Rector López Argüeta, 10871 Granada, Spain; ^3^Anthropological Institute & Museum, University of Zürich, Winterthurerstrasse 190, 8057 Zürich, Switzerland; ^4^Departamento de Ecología y Geología (Área de Paleontología), Facultad de Ciencias, Campus Universitario de Teatinos, 29071 Málaga, Spain; ^5^Departamento de Biología Molecular y Bioquímica, Facultad de Ciencias, Campus Universitario de Teatinos, 29071 Málaga, Spain

## Abstract

Brain volume and cheek-tooth size have traditionally been considered as two traits that show opposite evolutionary trends during the evolution of *Homo*. As a result, differences in encephalization and molarization among hominins tend to be interpreted in paleobiological grounds, because both traits were presumably linked to the dietary quality of extinct species. Here we show that there is an essential difference between the genus *Homo* and the living primate species, because postcanine tooth size and brain volume are related to negative allometry in primates and show an inverse relationship in *Homo*. However, when size effects are removed, the negative relationship between encephalization and molarization holds only for platyrrhines and the genus *Homo*. In addition, there is no general trend for the relationship between postcanine tooth size and dietary quality among the living primates. If size and phylogeny effects are both removed, this relationship vanishes in many taxonomic groups. As a result, the suggestion that the presence of well-developed postcanine teeth in extinct hominins should be indicative of a poor-quality diet cannot be generalized to all extant and extinct primates.

## 1. Introduction

Brain volume and tooth size, particularly those aspects concerning the dimensions of the postcanine dentition, have long been thought as crucial for hominin evolution [1–7]. It is widely accepted that, despite outstanding exceptions (e.g., *Homo floresiensis*), brain volume increased and postcanine tooth size decreased through the evolution of *Homo*. This negative correlation over time is particularly evident when the earliest members of the genus (i.e., *H. habilis* and *H. rudolfensis*) are compared with the geologically youngest species (i.e., *H. neanderthalensis* and *H. sapiens*), as the former have larger postcanine teeth and smaller brains, which allows describing a cladogenetic trend for the genus *Homo* [2–4, 7–9]. However, this trend is not observed among the species of australopiths, which showed small variations in tooth size and brain volume. Around 2.5 million years ago, two hominin lineages representing different adaptive strategies and showing a different relationship between tooth size and brain volume emerged: *Homo* and *Paranthropus*. Although postcanine tooth area differed substantially between the gracile and robust australopiths, with some species of *Paranthropus* showing a noticeable increase in tooth size (a phenomenon known as megadontia), this genus did not evidence a significant increase in brain mass or body size compared to *Australopithecus.* Therefore, during the course of hominin evolution different lineages changed independently in body size, tooth dimensions, and brain mass compared to an *Australopithecus*-like ancestor. For this reason, in spite of the existence of opposite trends for brain volume and postcanine tooth size in *Homo, *it seems that such inverse relationship would not probably hold statistically if all hominin taxa are considered jointly. This would mean that the coupled reduction of postcanine tooth size and brain expansion characteristic of the lineage that led to *H. sapiens* cannot be generalized to all hominins.

Brain volume has a direct reflection in cognitive abilities [10–12] and there is a well-known link between brain size and metabolic expenditure [1, 13–16]. For this reason, ecophysiological adaptations have long been proposed for explaining differences in brain size among primate species [17–19] and a number of researchers have suggested compensatory mechanisms for trading-off the high metabolic costs derived from an increase in encephalization, including changes in dietary quality and body composition [1, 8, 20–30].

Evolutionary changes in the dietary preferences of extinct hominins have been inferred from changes in the dimensions of the postcanine dentition, as cheek teeth are involved in food processing in the mouth. For this reason, the marked differences in tooth size between the robust australopithecines and both *Homo* and *Australopithecus africanus* were interpreted by Robinson as indicative of distinct adaptive strategies [31]. Robinson's “dietary hypothesis” considered the megadont teeth and enlarged areas of insertion for the temporalis and masseter muscles of *Paranthropus* as adaptations for a specialized diet consisting of hard and/or low-quality foodstuffs, whereas the craniodental architecture of *Australopithecus* was interpreted as evidencing more “generalist” (i.e., omnivorous) dietary habits. This enormously influential hypothesis has been supported by a number of studies of craniofacial functional morphology (e.g., see [32]) and also by patterns of dental microwear and carbon-isotopes ratios in tooth enamel (e.g., see [33, 34]). As a consequence, the presence of large cheek teeth uses to be interpreted as indicating the predominance of low-quality foodstuffs in both the living primates [35–39] and the extinct hominins [8, 31, 33, 40–42]. However, the statistical significance of the relationship between postcanine tooth size and a more direct estimate of dietary quality has not been adequately tested in primates. In addition, it has been proposed that postcanine tooth size reduction should not be envisioned as a process resulting exclusively from an increase in dietary quality, because it is related in nonhuman primates to a number of factors (e.g., toughness, hardness, and other physical properties of foodstuffs) [43, 44] and with changes in dietary preferences in the case of the extinct hominins [45], including the advent of advanced food processing techniques in *Homo erectus*, which resulted in a reduction of feeding time and a higher caloric intake [46].

This relationship is particularly important, because dietary changes (and, more specifically, an increase in dietary quality) could be involved in the negative correlation between the development of postcanine teeth and neurocranium volume. From the publication of the influential “expensive-tissue hypothesis” by Aiello and Wheeler [1], many studies have focused on searching for a direct relationship between brain size and dietary quality in primates [23–29, 47, 48]. However, no attempt for estimating the statistical relationship between postcanine tooth size and dietary quality has been carried out. In addition, it is not unreasonable to conceive a feedback between molarization and encephalization in hominins, because the presence of well-developed chewing teeth would be less necessary as long as larger brains would enhance extraoral processing (e.g., by tool makingand fire use) of foodstuffs that would otherwise be biomechanically unavailable [44, 46, 49]. This probably allowed early *Homo* to use a novel ecological niche, not exploited before by other primates and the australopiths, which involved the consumption of ungulate carcasses obtained through scavenging [50, 51]. Such dietary change opened up a new source of selective pressures for brain enlargement, favoring an increase in sociability, group size, and home range, which prompted the colonization of regions further from the equator, including the first human dispersal in Eurasia [50–53]. Under these circumstances, to invest metabolic energy in synthesizing unnecessary large teeth would have been penalized by natural selection.

However, larger primates tend to have larger brains and larger teeth than do smaller bodied ones. For this reason, and given the trend to body size increase in the course of hominin evolution, it is necessary to rule out that the negative correlation between molarization and encephalization of *Homo* merely emerges as an indirect consequence of the difference in scaling exponents for cheek-tooth area and brain volume on a third variable, body mass. As pointed out by Pilbeam and Gould [5], a relationship cannot be judged as adaptive until the changes in shape that result from size variations (i.e., allometric effects) are separated from those that reflect a specific adaptation.

It is well known that brain mass and postcanine tooth size follow specific allometric relationships with body mass, which depend on the taxonomic groupings analyzed (see reviews in [54] for brain mass and [55] for postcanine tooth size, resp.). For this reason, it is also important to control for phylogeny in the study of the relationships of molarization with dietary quality and with encephalization, in order to evaluate how these relationships scale in the taxonomic groupings considered and, thus, to estimate the extent to which such relationships are phylogenetically independent.

Consequently, this paper has two main goals: (1) to study in a sample of living primate species if there is a statistically significant, intertaxa allometric relationship between postcanine tooth size and dietary quality, as it has long been presumed that those species with poor-quality diets would require larger postcanine teeth and *vice versa*, and (2) to analyze the nature of the intertaxa allometric relationship between molarization and encephalization, evaluating if it emerges from the independent relationships of brain size and postcanine tooth size with body mass and also from phylogeny in the case of nonhuman primates.

## 2. Materials and Methods

In order to evaluate in a comparative context the evolutionary trend for brain volume and postcanine tooth size, 56 living primate species (Table S1) and 17 taxa of australopiths and *Homo* (Table S2) were studied. All data were collected from the literature (for further information, see Tables S1 and S2). The relationship was tested at different taxonomic groupings: all primates (including fossil hominins), nonhuman primates (excluding fossil hominins), prosimians, anthropoids, platyrrhines, catarrhines, cercopithecoids, hominoids, hominins, and *Homo*. In these comparisons, extinct hominins were not included within anthropoids, catarrhines, or hominoids. In the same way, *H. floresiensis* was excluded from some statistical analyses because this species represents a special case: it has disproportionally large teeth for its body size, as in the case of extant pygmies [56], and its brain is smaller than expected, as happens in insular dwarfs [57].

The variables used include the upper postcanine tooth area (PCTA, in mm^2^), estimated as the cumulative occlusal areas of P^4^, M^1^, and M^2^; brain mass (BrM, in g); body mass (BM, in kg); and dietary quality (DQ) (for additional details and bibliographic sources, see Tables S1 and S2). The last variable was defined as follows:

(1) $$\begin{array}{c} \text{DQ}=1\alpha +2\beta +3.5\gamma , \end{array}$$

where *α* is the percentage of leaves and structural parts of plants in the diet, *β* is the percentage of fruit and reproductive parts of plants (including nuts and seeds), and *γ* is the percentage of animal items (for more details, see SI).

In order to achieve normality, all variables were log-transformed prior to statistical analyses. Linear regression functions were adjusted using two methods, Reduced Major Axis (RMA) and Ordinary Least Squares (OLS). Regressions adjusted by OLS assume that the independent variable (*X*) is measured without error. However, it should be noted that body masses are estimated for extinct hominins. Size effects were removed adjusting OLS separate regressions functions of PCTA, BrM, and DQ on BM. Then, the residuals of the dependent variables (PCTA_BMres_, BrM_BMres_, and DQ_BMres_) were used for obtaining “size-free” adjustments. DQ effects on BrM and PCTA and BrM effects on PCTA and DQ were avoided following a similar procedure.

Given that the species analyzed are part of a hierarchically structured phylogeny, data collected from them do not necessarily satisfy the condition of statistical independence, thus hindering traditional (i.e., ahistorical) statistical analyses [58, 59]. This may translate in spuriously narrow confidence limits for the statistics used (i.e., increased type I error) and inaccurate estimates of the parameters of interest [59]. Both problems, however, can be largely circumvented by including phylogenetic information into the statistical analyses, which allows obtaining phylogenetically independent data. For this reason, the original variables were transformed into their corresponding contrasts according to Phylogenetic Generalized Least Squares (PGLS) using COMPARE 4.6b [60], following the procedure described by Martins and Hansen [61]. In brief, a simple exponential model of constrained phenotypic evolution is applied, with an estimated alpha parameter (*α*) used to indicate the strength of the evolutionary constraint. When both within-species variation and evolutionary constraint are close to zero, PGLS results will be identical to those produced using Felsenstein's independent contrasts method (FIC). For details on the procedure used, see SI.

Composite trees, including branch length estimates, were developed for reconstructing the phylogenetic relationships among the primate species studied, following the consensus tree from 10KTrees Project (Primates) (Figure S1) [62].

## 3. Results

The present study does not focus separately on either molarization or encephalization nor on the relationships of dietary quality with body mass and with brain mass. However, we were forced to control for body mass and therefore to study how brain mass, cheek-tooth area, and dietary quality change with body size.

First, it is worth noting that the results obtained do not differ according to the regression method used (i.e., RMA or OLS) (Tables S3 and S4), except for all primates and catarrhines in the regression of logPCTA-logBrM (Table S4). In the case of primates, isometry may be discarded when OLS is used, but not for RMA. In the case of catarrhines, isometry is rejected with RMA and not with OLS. Thus, in general terms we can affirm that the results obtained are not sensitive to the method of adjustment used. For this reason, only the regressions adjusted by OLS are described in this section (see SI for complete regression statistics).

Secondly, although Gould [63, 64] predicted that the slopes obtained for major taxonomic groupings would be greater than the ones adjusted for lower rank taxa, the results of this study do not support such a trend for postcanine tooth size and brain mass (Table S4): the slopes for the most inclusive taxonomic grouping (i.e., nonhuman primates) take values of 0.68 (RMA) and 0.56 (OLS), while in all taxonomic groupings of lower rank the values are close to one.

**Figure 1:**
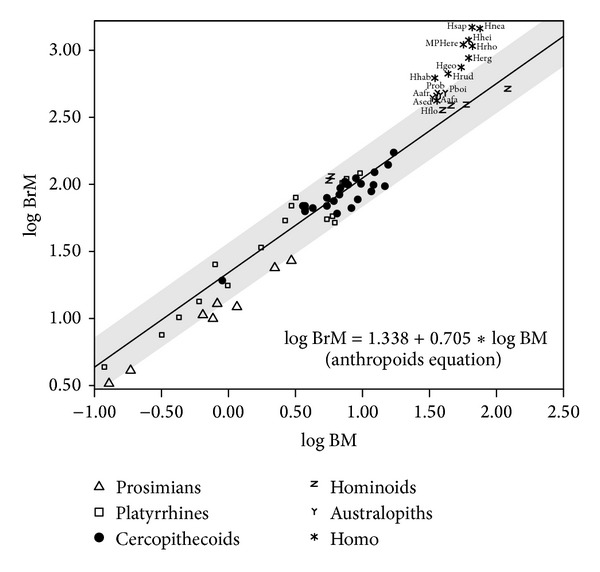
Bivariate plot for the logarithms of body mass (BM, in kg) and brain mass (BrM, in g). The regression line and its 95% confidence interval (grey shadow) were adjusted with anthropoids. Aafa: *A. afarensis*, Aafr: *A. africanus*, Ased: *A. sediba*, Hflo: *Homo floresiensis*, Herg: *H. ergaster*, Hgeo: *H. georgicus*, Hhab: *H. habilis*, Hhei: *H. heidelbergensis*, Hnea: *H*. *neanderthalensis*, Hrod: *H. rhodesiensis*, Hrud: *H. rudolfensis*, Hsap: *H. sapiens*, MPHerec: Middle Pleistocene *H. erectus*, Pboi: *Paranthropus boisei*, Prob: *P. robustus*.

In the case of the regressions of brain mass on body mass, the slopes obtained for the different groupings and taxonomic levels considered are all significantly lower than one, with the only exception of the categories extinct hominins and *Homo*, which take values close to 1.5. Several taxonomic subsets (all primates, prosimians, cercopithecoids, extinct hominins and *Homo*) show slopes that are statistically different from 0.75 (Figure 1; Table S3, upper part), the value expected from interspecific scaling of metabolic requirements on body mass in both mammals [65–68] and primates [69, 70]. In addition, when the relationship between brain size and body mass is analyzed in a “taxon-free” approach (i.e., discarding the effects of phylogeny), a slope close to 0.75 and a high correlation value are maintained (Table S6).

**Figure 2:**
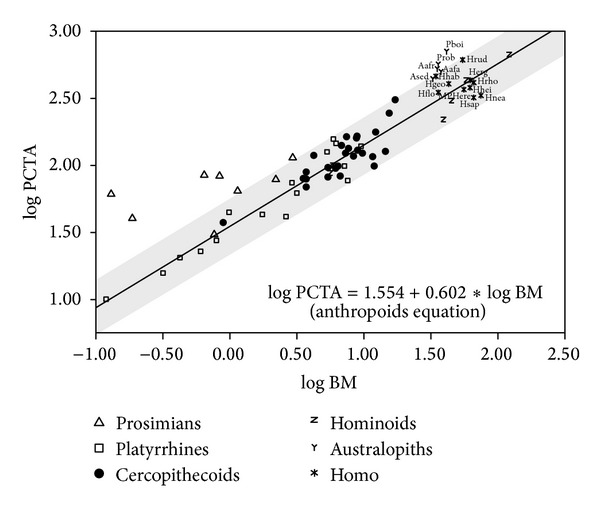
Bivariate plot for the logarithms of body mass (BM) and postcanine tooth area (PCTA, in mm^2^). The regression line and its 95% confidence interval (grey shadow) were adjusted with anthropoids. For species abbreviations, see legend of Figure 1.

The relationship between postcanine tooth area and body mass provides quite diverse results in the primate groups analyzed (Figure 2; Table S3, lower part). Some taxonomic groupings (e.g., all primates, anthropoids, platyrrhines, and catarrhines) are in agreement with the expectations from isometry (i.e., the obtaining of a slope close to 2/3) and disagree with the expectations from metabolic scaling (i.e., an exponent of 0.5). In contrast, there are groups (e.g., cercopithecoids and hominoids) in which the slopes obtained are consistent with those expected from both metabolic scaling and isometry, while in all primates the result is the opposite of the one predicted. In some cases, these results are coherent with those reported previously, but in others they are in clear disagreement [5, 35–38, 55, 71–81]. The ambiguity of these results probably indicates that the slopes obtained vary as a function of the variables and samples used here and in previous studies (see [55] for a comprehensive compilation). When phylogeny is controlled, the relationship between body mass and postcanine occlusal area disappears (Table S6).

**Figure 3:**
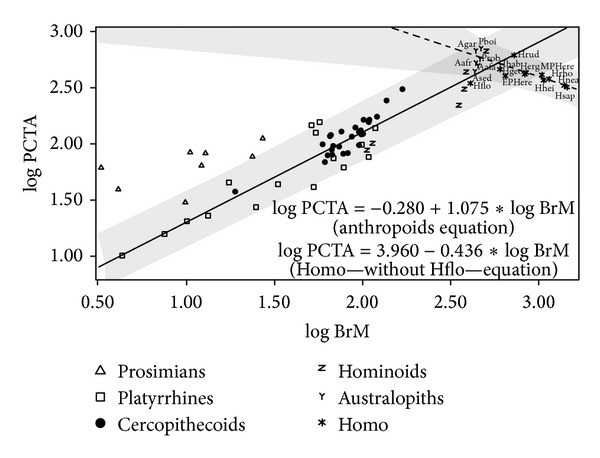
Bivariate plot for the logarithms of brain mass (BrM) and postcanine tooth area (PCTA). The solid regression line and its 95% confidence interval (grey shadow) were adjusted with anthropoids and the dashed line and its 95% confidence interval (grey shadow) were adjusted with *Homo* excepting *H. floresiensis*. Agar: *A. garhi*, EPHerec: Early Pleistocene *H. erectus*. For the remaining species abbreviations, see legend of Figure 1.

**Figure 4:**
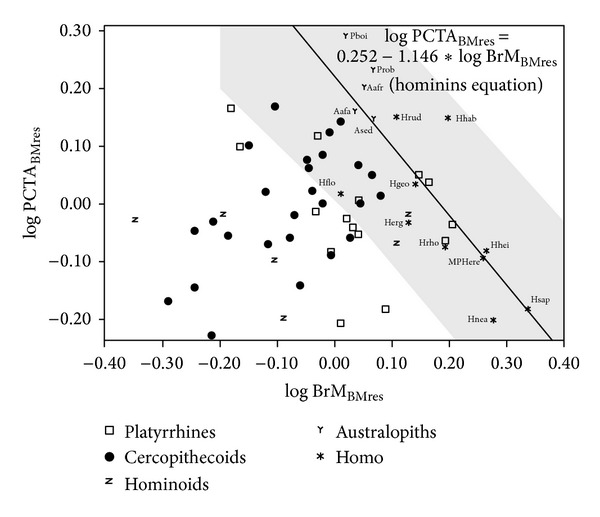
Bivariate plot of the residuals of postcanine tooth area (PCTA) on body mass (BM) over the residuals of brain mass (BrM) on BM. The regression line and its 95% confidence interval (grey shadow) were adjusted with data for fossil hominins. For species abbreviations, see legend of Figures 1 and 3.

**Figure 5:**
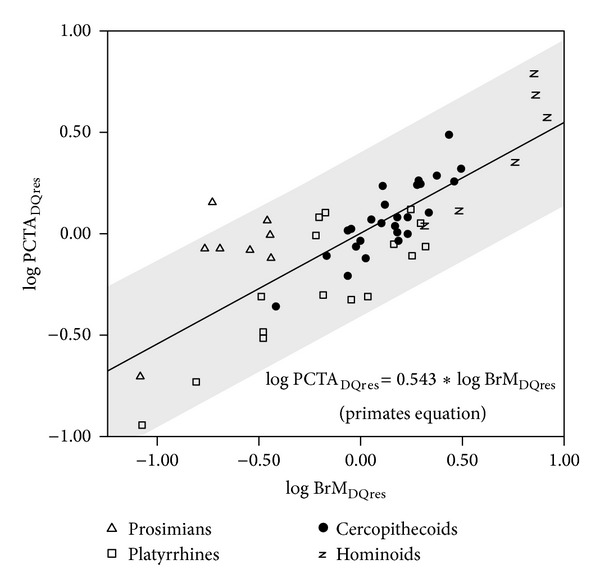
Bivariate plot of the residuals of postcanine tooth area (PCTA) on dietary quality (DQ) over the residuals of brain mass (BrM) on DQ. The regression line and its 95% confidence interval (grey shadow) were adjusted with data for nonhuman primates.

Table S4 (upper part) and Figure 3 summarize the results of the regressions of postcanine tooth area on brain mass. With the only exception of prosimians and *Homo*, the slopes for all other groupings are statistically different from zero. However, when *H. floresiensis* is removed, the slope adjusted for *Homo* is statistically different from zero. The null hypothesis of isometry cannot be ruled out for nonhuman primates, platyrrhines, catarrhines, and hominoids, but isometry is rejected for primates, which show negative allometry, and for anthropoids and cercopithecoids, which all scale with positive allometry. In contrast, the adjustments for extinct hominins and for *Homo* without *H. floresiensis* show negative slopes that follow the expectations from inverse proportionality (i.e., a slope close to −1). Thus, although there is a significant relationship between brain mass and postcanine tooth area, it depends on the taxonomic groups analyzed, as in the case of body mass. As a consequence, the inverse relationship between brain volume and tooth size found in the genus *Homo* cannot be extrapolated to all living primates. However, when differences in body mass are discarded (Figure 4; Table S4, middle part), the only taxonomic groupings with slopes that are statistically different from zero are prosimians, which show a positive slope, and anthropoids, platyrrhines, extinct hominins, and *Homo,* which all take negative slopes. However, it is worth noting that when australopiths are analyzed separately from other extinct hominins, they do not show an inverse relationship. This means that the negative slope found for all extinct hominins results from the inclusion of *Homo*. If the effects of dietary quality are avoided using the residuals of the equation obtained by OLS for nonhuman primates, the most inclusive grouping, all other taxonomic groupings show correlations that are statistically different from zero (Figure 5; Table S4, lower part). Except for nonhuman primates, which show negative allometry, all other groupings take values that do not allow discarding isometry. As concerns the metabolic exponent (0.5), it may not be rejected for nonhuman primates, prosimians, platyrrhines, and hominoids. If the effects of phylogeny are controlled (Table S6), the whole set of primate species analyzed shows a statistically significant isometric relationship between the two variables cited above. However, when the effects of body mass and phylogeny are both discarded, the relationship between these variables vanishes (Table S6).

The distribution of hominin taxa on the morphospace defined by the logarithms of brain mass and postcanine tooth area (Figure 3) shows that the gracile and robust australopiths plot on the region between the regression line for anthropoids and its upper 95% confidence limit. This indicates that australopiths had larger cheek teeth than expected for an anthropoid of similar brain size. In contrast, Early Pleistocene *Homo* and *H. floresiensis* scatter on the region between the regression line and its lower 95% confidence limit, which shows the opposite condition. Finally, all Middle and Upper Pleistocene *Homo*, except *H. floresiensis*, lie in the region situated below the lower confidence limit, which is a reflection of the inverse trends for encephalization and molarization in the evolution of *Homo*. A similar morphometric pattern is maintained when the effects of body size are removed (Figure 4).

**Figure 6:**
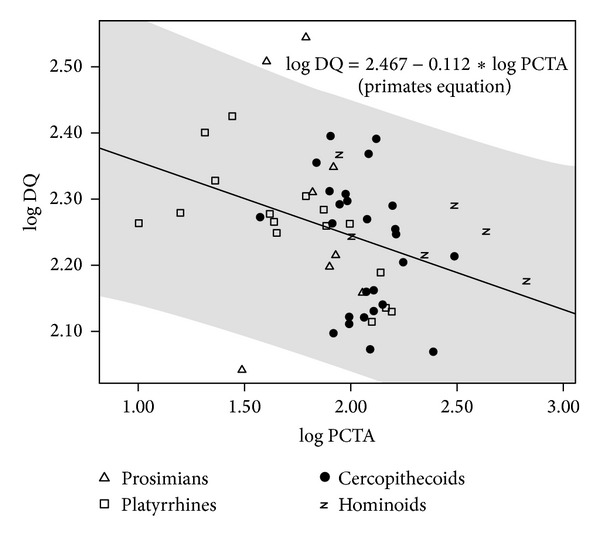
Bivariate plot of dietary quality (DQ) on postcanine tooth area (PCTA). The regression line and its 95% confidence interval (grey shadow) were adjusted with data for nonhuman primates.

**Figure 7:**
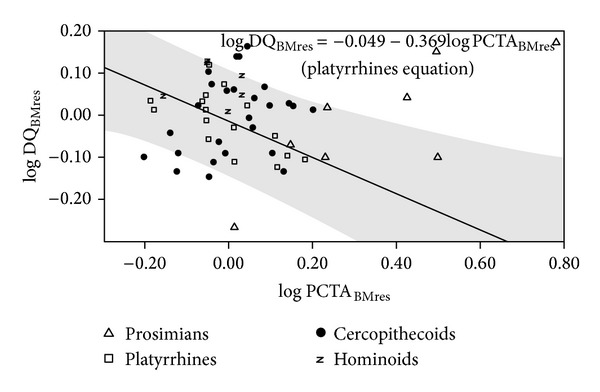
Bivariate plot of the residuals of dietary quality (DQ) on body mass (BM) over the residuals of postcanine tooth occlusal area (PCTA) on BM. The regression line and its 95% confidence interval (grey shadow) were adjusted with data for platyrrhines.

**Figure 8:**
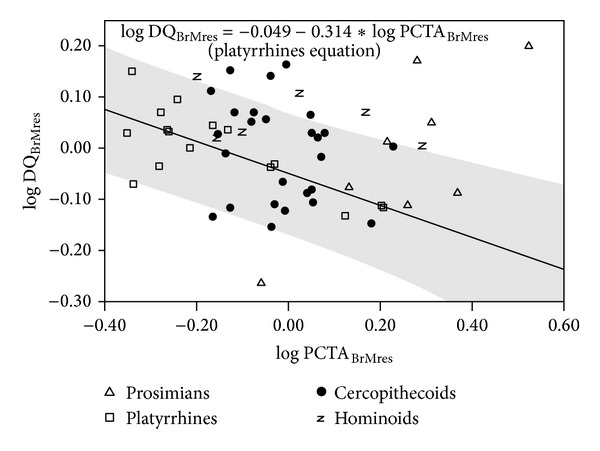
Bivariate plot of the residuals of dietary quality (DQ) on brain mass (BrM) over the residuals of postcanine tooth occlusal area (PCTA) on BrM. The regression line and its 95% confidence interval (grey shadow) were adjusted with data for platyrrhines.

A number of regressions were adjusted for evaluating the statistical relationships between postcanine tooth area and dietary quality (Table S5). Negative slopes were obtained in some taxonomic groupings such as nonhuman primates, anthropoids, and platyrrhines (Figure 6; Table S5, upper part). However, when the regressions were size-adjusted, statistically significant slopes were obtained only for prosimians, positive slope, and platyrrhines as the only group showing a negative slope (Figure 7; Table S5, middle part). Again, this indicates that there is no general rule for the different subsets of extant primates. In any case, the result most commonly obtained is the absence of statistical significance when size effects are detracted. In addition, regression analyses were performed for evaluating the relationship between postcanine dentition and dietary quality when brain size effects are removed (Figure 8; Table S5, lower part). Results were similar to those obtained when body size effects were removed, as the only two groups that provide statistically significant slopes were prosimians and platyrrhines, as well as anthropoids. Finally, the statistical significance of the negative relationship between postcanine tooth area and dietary quality found in nonhuman primates holds after phylogenetic correction but is lost when phylogeny and size effects are both removed and also when the effects of phylogeny and brain size are discarded (Table S6).

## 4. Discussion

The relationship between postcanine tooth size, metabolic requirements, and dietary preferences is an old topic that periodically experiences a renewed interest in both primatology [35–39, 55] and paleoanthropology [29–33, 40–42, 82]. Specifically, there is a persistent debate on whether the dimensions of postcanine teeth scale isometrically with the size of the animals or evidence their metabolic requirements, which have been assumed to scale to the 0.75 power of body mass (Kleiber's law). However, it is worth noting that a number of recent studies have pointed out that no pure power law describes the scaling of energetic demands on body mass and, consequently, that it is not possible to predict a theoretical value for metabolic scaling [54, 70, 83–87]. This is not a major problem in the case of primates, because a power law with an exponent of 3/4 can be considered as a reasonable proxy even when phylogenetic effects are removed ([69, 70], this analysis).

One common feature of previous studies is that they rely on indirect inferences, basically those derived from the “equal exponent” hypothesis for the scaling of tooth size and body mass. For example, several researchers have proposed that cheek-tooth area reflects the energetic demands of primates, because the values obtained for the slope of the bilogarithmic regression between tooth size and body mass are positive [5, 38, 71, 73, 75, 78–81]. However, as McNab and Eisenberg pointed out, the mere equivalence of exponents is by itself inadequate for inferring a functional link [88] and can lead to erroneous conclusions. In other words, if two dependent variables show similar slopes when they are regressed on the same independent variable, this does not necessarily imply a direct functional link between both variables. In addition, to consider that the finding of a slope of ~0.75 between postcanine tooth area (a two-dimensional variable) and body mass (a three-dimensional one) reflects positive allometry is based on an erroneous assumption, as it does not consider the difference in the orders of magnitude of both variables. This should only apply if 0.67 (i.e., the isometric exponent) is multiplied by 0.75, as this would result in a value of 0.5. For this reason, the direct relationship between postcanine tooth area and basal metabolic rate was tested recently [89], showing a strong correlation between both variables with independence of body size and phylogeny.

The metabolic interpretation of postcanine tooth area is based on functional grounds, as cheek teeth are used for chewing food before swallowing. For this reason, it has long been proposed that those species adapted to a poor diet (i.e., one containing a high amount of fibrous foods and/or leaves, which are difficult to digest) will show cheek teeth with a well-developed occlusal surface [90]. In addition, in the case of extinct hominins technology played a relevant role in the reduction of the postcanine teeth, as the marked decrease in molar size shown by *H. erectus* was probably related to advances in extraoral food processing [30, 46].

The results obtained in this study do not clarify if there is an isometric or metabolic scaling for postcanine tooth area on body mass, because no common, unambiguous pattern was obtained for all primate species. This agrees with the absence of a universal pattern of scaling for tooth size on body mass in mammals [44]. However, it is obvious that there are differences between the living primates and the extinct hominins, because tooth size is related to body mass with negative allometry in the former and does not show any relationship in the latter. Although this is probably due to the huge range of error associated with the mass estimates of extinct hominins, such findings suggest that the special features of the genus *Homo* preclude the use of other primate taxa for drawing analogies in order to infer functional or developmental constraints.

The lack of parallelism between *Homo* and other primates is more obvious in the relationship between brain mass and postcanine tooth area. Unlike most primate groups analyzed, and specifically the closest taxa (i.e., catarrhines, cercopithecoids, and hominoids), an inverse relationship between brain mass and tooth size holds after correction for body mass differences in the species of the genus *Homo* when *H. floresiensis* is removed from the data set. This allows rejecting the hypothesis that molarization and encephalization correlate indirectly in *Homo* via their correlation with body mass. In fact, an inverse relationship between brain mass and postcanine tooth area with independence of body mass is only found in platyrrhines among the living primates. In addition, the fact that brain volume and postcanine tooth area correlate positively in nonhuman primates after removing dietary effects implies that differences in dietary quality among the living primates cannot be used as a model for explaining changes in encephalization and molarization during the evolution of the genus *Homo.* For this reason, the “expensive-tissue hypothesis,” which assumes that the metabolic requirements of the enlarged human brain were offset by a corresponding reduction of the gut and resulted in a dietary shift to high-quality, easy-to-digest foodstuffs such as meat [1], would be only applicable to the genus *Homo.* In fact, a recent study of one hundred mammalian species (including 23 primates) has shown that brain size is not negatively correlated with the mass of the digestive tract, which refutes the expensive-tissue hypothesis for mammals [91].

In this way, the size of the postcanine dentition is related to dietary quality with independence of brain size in the case of prosimians and platyrrhines. This is also the case for anthropoids, although the correlation is not significant for catarrhines. For this reason, we can conclude that the relationship detected in anthropoids emerges because they include platyrrhines. In any case, such relationship is biased by phylogeny. For this reason, from the results obtained in this study we can affirm that the relationship between tooth size and dietary quality is, at least partially, dependent on brain size in nonhuman primates.

Therefore, if dietary quality, including cooking and nonthermal, extraoral food processing [46], is the functional link that explains the opposite trends for molarization and encephalization shown by the members of *Homo*, it would be necessary to find a mechanism that operates in this genus and not in other primates and mammals. In this context, two hypotheses must be taken into account.

The first considers the relative development of the temporal muscles as such causal link. In fact, given that the dimensions of the chewing teeth and of the masticatory muscles scale isometrically in hominins [92], a change in the size of the chewing muscles might imply a change of the same magnitude in the postcanine dentition, which would mean that both traits are functionally integrated. Stedman et al. [93] have proposed that MYH16 gene inactivation was the factor that resulted in an increase of encephalization in *Homo* by eliminating the constraints posed by a well-developed temporal musculature on braincase development. In other words, if there were morphogenetic limitations that determine an inverse relationship between tooth size and neurocranium volume, and if such constraints were subsequently removed, this might mean that a new, previously nonexistent combination of traits (i.e., tooth reduction and brain expansion) could arise, which would be favored later by natural selection. In fact, it is not unreasonable to conceive a certain feedback between encephalization and molarization in *Homo*, because an enlarged brain would open the possibility of tool use for extraoral processing of foodstuffs. This would make the presence of well-developed chewing teeth less necessary, which would allow saving the metabolic energy required for synthesizing them. An essential element of this hypothesis is that the age of the gene inactivation event (~2.4 Ma) [94] roughly coincides with the first appearance of the genus *Homo* and also with the earliest evidence of tool making in the archaeological record. However, it is worth noting that Perry et al. [94] estimated the age of the deletion at ~5.3 Ma, which would invalidate the inactivation of MYH16 gene as the key factor for the encephalization of *Homo*. In addition, McCollum et al. [95] have argued that, according to current knowledge of neurocranial growth and development, it is unlikely that MYH16 gene mutation would have led to dramatic changes in the masticatory mechanics of early hominins and thus in the craniofacial evolution of *Homo*.

The second hypothesis arises from a recent discovery. Inhibition of SRGAP2 gene function by its human-specific paralogs (SRGAP2C) has contributed to the evolution of the human neocortex and plays an important role during human brain development [96]. Such transcendental change occurred at the same time than inhibition of MYH16 gene. Therefore, from a genetic point of view it could be considered that the synchronization of brain enlargement and postcanine tooth size reduction was not merely coincidental.

The results of our study show that although there is a significant relationship between postcanine tooth area and dietary quality in some taxonomic groupings, even after phylogenetic correction, in most cases this correlation vanishes when the effects derived from differences in body mass are removed. Prosimians and platyrrhines are the only exception. In the case of prosimians, our results agree with those of Vinyard and Hanna [81], who showed that the species with higher quality diet have more developed postcanine teeth. This means that New World monkeys are the only living nonhuman primates that provide direct empirical evidence for those aspects of the “dietary hypothesis” that are related to postcanine tooth area, as the species of platyrrhines with poorer diet show relatively larger cheek teeth. Obviously, this fact does not imply that Robinson's “dietary hypothesis” is not applicable to the extinct hominins, but it means that such interpretive context should not be generalized to all extant and extinct primates. Therefore, a universal relationship between tooth size and dietary quality seems improbable. In addition, recent research on hominin diets has even shown that the large teeth of *P. boisei* were used to process foodstuffs with quite divergent mechanical properties. This has led to the hypothesis that tooth morphology does not reflect the most commonly masticated items but sometimes reflects “fallback” food items, which are rarely consumed but ultimately important for the organism's survival [97].

Two issues must be raised for explaining particular aspects of several taxa included in this study. The first is the anomalous position of *H. floresiensis*, which is positioned in Figure 4 near the limit of the confidence interval for hominins. This may be explained by two patterns observed in dwarf populations. Specifically, African and Filipino pygmies show teeth larger than expected for their body masses. This is related to low levels of insulin-like growth factor I (IGF-I), which affects body size (including brain size) and not tooth size [56]. The other pattern is evidenced by *Myotragus balearicus*, an insular bovid with a reduced brain that may be explained by absence of predators and food limitations [57]. The second issue concerns the Early Pleistocene remains from Dmanisi (Georgia). This human population, the oldest one that was found out of Africa (1.85 My) [98], has been described as an example of mosaic evolution, because it shows a derived postcranial skeleton [99], teeth that resemble those of contemporary African populations but showing also features found in younger taxa [100], relatively small body size, and a proportion between the two main cranial modules (i.e., splanchnocranium and neurocranium) that resembles the one found in early *Homo* [53]. Our results confirm such inferences, because this Georgian population has a relationship between tooth size and endocranium volume, which is similar to the one of *H. habilis s.s.*, and takes an intermediate value between *H. habilis* and *H. ergaster* when body size effects are removed. For this reason, if a change in dietary habits (and, more specifically, an increase in dietary quality resulting from an increase in meat and fat consumption) was related to the first human dispersal out of Africa, such change took place soon after the emergence of the genus *Homo*.

Whatever it takes, a number of aspects not considered in this study could be also influencing the relationship between tooth size and dietary quality, including the morphology of the dental cusps, the mechanical properties of food (external and internal), or the amount of food that is processed simultaneously into the mouth [83]. These features will be the object of future studies.

## Supplementary Material

Additional tables and figures, with further discussion of materials and methods as well as additional references not included in the main text.

## References

[1] Aiello LC, Wheeler P (1995). The expensive-tissue hypothesis: the brain and the digestive system in human and primate evolution. *Current Anthropology*.

[2] Klein RG (1989). *The Human Career*.

[3] McHenry HM (1982). The pattern of human evolution: studies on bipedalism, mastication, and encephalization. *Annual Review of Anthropology*.

[4] McHenry HM, Coffing K (2000). *Australopithecus* to *Homo*: transformations in body and mind. *Annual Review of Anthropology*.

[5] Pilbeam D, Gould SJ (1974). Size and scaling in human evolution. *Science*.

[6] Weidenreich F (1941). The brain and its rôle in the phylogenetic transformation of the human skull. *Transactions of the American Philosophical Society*.

[7] Wood BA, Collard M (1999). The human genus. *Science*.

[8] Leonard WR, Snodgrass JJ, Robertson ML (2007). Effects of brain evolution on human nutrition and metabolism. *Annual Review of Nutrition*.

[9] McHenry HM (1984). Relative cheek-tooth size in *Australopithecus*. *American Journal of Physical Anthropology*.

[10] Deaner RO, Isler K, Burkart J, van Schaik C (2007). Overall brain size, and not encephalization quotient, best predicts cognitive ability across non-human primates. *Brain, Behavior and Evolution*.

[11] Jerison HJ (1955). Brain to body ratios and the evolution of intelligence. *Science*.

[12] Jerison HJ (1973). *Evolution of the Brain Size and Intelligence*.

[13] Armstrong E (1983). Relative brain size and metabolism in mammals. *Science*.

[14] Isler K, Kirk EC, Miller JMA, Albrecht GA, Gelvin BR, Martin RD (2008). Endocranial volumes of primate species: scaling analyses using a comprehensive and reliable data set. *Journal of Human Evolution*.

[15] Martin RD (1981). Relative brain size and basal metabolic rate in terrestrial vertebrates. *Nature*.

[16] Martin RD (1983). *Human Brain Evolution in an Ecological Context: 52th James Arthur Lecture on the Evolution of the Human*.

[17] Clutton-Brock TH, Harvey PH (1980). Primates, brains and ecology. *Journal of Zoology*.

[18] Foley RA, Lee PC, Widdowson EM, Knight CD, Jonxis JHP (1991). Ecology and energetics of encephalization in hominid evolution. *Philosophical Transactions of The Royal Society B*.

[19] Harvey PH, Clutton-Brock TH, Mace GM (1980). Brain size and ecology in small mammals and primates. *Proceedings of the National Academy of Sciences of the United States of America*.

[20] Fish JL, Lockwood CA (2003). Dietary constraints on encephalization in primates. *American Journal of Physical Anthropology*.

[21] Isler K, van Schaik C (2006). Costs of encephalization: the energy trade-off hypothesis tested on birds. *Journal of Human Evolution*.

[22] LemaÎtre J-F, Ramm SA, Barton RA, Stockley P (2009). Sperm competition and brain size evolution in mammals. *Journal of Evolutionary Biology*.

[23] Leonard WR (2002). Food for thought. Dietary change was a driving force in human evolution. *Scientific American*.

[24] Leonard WR, Robertson ML (1992). Nutritional requirements and human evolution: a bioenergetics model. *American Journal of Human Biology*.

[25] Leonard WR, Robertson ML (1994). Evolutionary perspectives on human-nutrition: the influence of brain and body-size on diet and metabolism. *American Journal of Human Biology*.

[26] Leonard WR, Robertson ML (1997). Comparative primate energetics and hominid evolution. *American Journal of Physical Anthropology*.

[27] Leonard WR, Ulijaszek SJ (2002). Energetics and evolution: an emerging research domain. *American Journal of Human Biology*.

[28] Leonard WR, Robertson ML, Aiello LC, Wheeler P (1996). On diet, energy metabolism, and brain size in human evolution. *Current Anthropology*.

[29] Leonard WR, Robertson ML, Snodgrass JJ, Kuzawa CW (2003). Metabolic correlates of hominid brain evolution. *Comparative Biochemistry and Physiology A*.

[30] Snodgrass JJ, Leonard WR, Robertson ML, Hublin M Richards JJ (2009). The energetics of encephalization in early hominids. *The Evolution of Hominid Diets: Integrating Approaches to the Study of Palaeolithic Subsistence*.

[31] Robinson JT (1954). Prehominid dentition and hominid evolution. *Evolution*.

[32] Wood B, Strait D (2004). Patterns of resource use in early *Homo* and *Paranthropus*. *Journal of Human Evolution*.

[33] Grine FE (1986). Dental evidence for dietary differences in *Australopithecus* and *Paranthropus*: a quantitative analysis of permanent molar microwear. *Journal of Human Evolution*.

[34] Grine FE, Sponheimer M, Ungar PS, Lee-Thorp J, Teaford MF (2012). Dental microwear and stable isotopes inform the paleoecology of extinct hominins. *American Journal of Physical Anthropology*.

[35] Goldstein S, Post D, Melnick D (1978). An analysis of cercopithecoid odontometrics. I. The scaling of the maxillary dentition. *American Journal of Physical Anthropology*.

[36] Kay RF (1975). The functional adaptations of primate molar teeth. *American Journal of Physical Anthropology*.

[37] Pan R (2006). Dental morphometric variation between African and Asian colobines, with special reference to the other Old World monkeys. *Journal of Morphology*.

[38] Pirie PL (1978). Allometric scaling in the postcanine dentition with reference to primate diets. *Primates*.

[39] Strait SG (1993). Differences in occlusal morphology and molar size in frugivores and faunivores. *Journal of Human Evolution*.

[40] Garn SM, Lewis AB (1958). Tooth-size, body-size and “giant” fossil man. *American Anthropologist*.

[41] Wolpoff MH (1973). Posterior tooth size, body size, and diet in South African gracile *Australopithecines*. *American Journal of Physical Anthropology*.

[42] Kay RF (1985). Dental evidence for the diet of *Australopithecus*. *Annual Review of Anthropology*.

[43] Wright BW (2005). Craniodental biomechanics and dietary toughness in the genus *Cebus*. *Journal of Human Evolution*.

[44] Ross CF, Washington RL, Eckhardt A (2009). Ecological consequences of scaling of chew cycle duration and daily feeding time in primates. *Journal of Human Evolution*.

[45] de Castro JMB, Nicolás E (1995). Posterior dental size reduction in hominids: the Atapuerca evidence. *American Journal of Physical Anthropology*.

[46] Organ C, Nunn CL, Machanda Z, Wrangham RW (2011). Phylogenetic rate shifts in feeding time during the evolution of *Homo*. *Proceedings of the National Academy of Sciences of the United States of America*.

[47] Fish JL, Lockwood CA (2003). Dietary constraints on encephalization in primates. *American Journal of Physical Anthropology*.

[48] Allen KL, Kay RF (2011). Dietary quality and encephalization in platyrrhine primates. *Proceedings of the Royal Society B*.

[49] Wrangham R (2009). *Catching Fire: How Cooking Made Us Human*.

[50] Arribas A, Palmqvist P (1999). On the ecological connection between sabre-tooths and hominids: faunal dispersal events in the Lower Pleistocene and a review of the evidence for the first human arrival in Europe. *Journal of Archaeological Science*.

[51] Espigares MP, Martínez-Navarro B, Palmqvist P (2013). *Homo* vs. *Pachycrocuta*: earliest evidence of competition for an elephant carcass between scavengers at Fuente Nueva-3 (Orce, Spain). *Quaternary International*.

[52] Antón SC, Leonard WR, Robertson ML (2002). An ecomorphological model of the initial hominid dispersal from Africa. *Journal of Human Evolution*.

[53] Jiménez-Arenas JM, Palmqvist P, Pérez-Claros JA (2011). A probabilistic approach to the craniometric variability of the genus *Homo* and inferences on the taxonomic affinities of the first human population dispersing out of Africa. *Quaternary International*.

[54] Capellini I, Venditti C, Barton RA (2010). Phylogeny and metabolic scaling in mammals. *Ecology*.

[55] Copes LE, Schwartz GT (2010). The scale of it all: postcanine tooth size, the taxon-level effect, and the universality of Gould’s scaling law. *Paleobiology*.

[56] Shea BT, Gomez AM (1988). Tooth scaling and evolutionary dwarfism: an investigation of allometry in human pygmies. *American Journal of Physical Anthropology*.

[57] Köhler M, Moyà-Solà S (2004). Reduction of brain and sense organs in the fossil insular bovid *Myotragus*. *Brain, Behavior and Evolution*.

[58] Felsenstein J (1985). Phylogenies and the comparative method. *American Naturalist*.

[59] Harvey PH, Pagel MD (1991). *The Comparative Method in Evolutionary Biology*.

[60] Martins EP COMPARE, Computer programs for the statistical analysis of comparative data. http://compare.bio.indiana.edu.

[61] Martins EP, Hansen TF (1997). Phylogenies and the comparative method: a general approach to incorporating phylogenetic information into the analysis of interspecific data. *American Naturalist*.

[62] Arnold C, Matthews LJ, Nunn CL (2010). The 10kTrees website: a new online resource for primate phylogeny. *Evolutionary Anthropology*.

[63] Gould SJ (1966). Allometry and size in ontogeny and phylogeny. *Biological Reviews*.

[64] Gould SJ (1971). Geometric similarity in allometric growth: a contribution to the problem of scaling in the evolution of size. *American Naturalist*.

[65] Calder WA (1984). *Size, Function, and Life History*.

[66] Glazier DS (2005). Beyond the “3/4-power law”: variation in the intra- and interspecific scaling of metabolic rate in animals. *Biological Reviews*.

[67] Kleiber M (1961). *The Fire of Life: An Introduction to Animal Energetics*.

[68] Peters RH (1983). *The Ecological Implications of Body Size*.

[69] Genoud M (2002). Comparative studies of basal rate of metabolism in primates. *Evolutionary Anthropology*.

[70] White CR, Blackburn TM, Seymour RS (2009). Phylogenetically informed analysis of the allometry of mammalian basal metabolic rate supports neither geometric nor quarter-power scaling. *Evolution*.

[71] Corruccini RS, Henderson AM (1978). Multivariate dental allometry in primates. *American Journal of Physical Anthropology*.

[72] Gingerich PD (1977). Correlarion of tooth size and body size in living hominoid primates, with a note on relative brain size in *Aegyptopithecus* and *Proconsul*. *American Journal of Physical Anthropology*.

[73] Gingerich PD, Smith BH, Jungers WL (1985). Allometric scaling in the dentition of primates and insectivores. *Size and Scaling in Primate Biology*.

[74] Gingerich PD, Smith BH, Rosenberg K (1982). Allometric scaling in the dentition of primates and prediction of body weight from tooth size in fossils. *American Journal of Physical Anthropology*.

[75] Gould SJ (1975). On the scaling of tooth size in mammals. *American Zoologist*.

[76] Kanazawa E, Rosenberger AL (1989). Interspecific allometry of the mandible, dental arch, and molar area in anthropoid primates: functional morphology of masticatory components. *Primates*.

[77] Kay RF (1994). ‘Giant’ tamarin from the Miocene of Colombia. *American Journal of Physical Anthropology*.

[78] Kieser JA, Groeneveld HT (1990). Static intraspecific allometry of the dentition in *Otolemur crassicaudatus*. *Zoological Journal of the Linnean Society*.

[79] Smith RJ (1981). On the definition of variables in studies of primate dental allometry. *American Journal of Physical Anthropology*.

[80] Smith RJ (1996). Biology and body size in human evolution: statistical inference misapplied. *Current Anthropology*.

[81] Vinyard CJ, Hanna J (2005). Molar scaling in strepsirrhine primates. *Journal of Human Evolution*.

[82] Lucas PW, Constantino PJ, Wood BA (2008). Inferences regarding the diet of extinct hominins: structural and functional trends in dental and mandibular morphology within the hominin clade. *Journal of Anatomy*.

[83] Dodds PS, Rothman DH, Weitz JS (2001). Re-examination of the “3/4-law” of metabolism. *Journal of Theoretical Biology*.

[84] Kolokotrones T, van Savage VS, Deeds EJ, Fontana W (2010). Curvature in metabolic scaling. *Nature*.

[85] Lovegrove BG (2000). The zoogeography of mammalian basal metabolic rate. *American Naturalist*.

[86] Sieg AE, O’Connor MP, McNair JN, Grant BW, Agosta SJ, Dunham AE (2009). Mammalian metabolic allometry: do intraspecific variation, phylogeny, and regression models matter?. *American Naturalist*.

[87] Symonds MRE, Elgar MA (2002). Phylogeny affects estimation of metabolic scaling in mammals. *Evolution*.

[88] McNab BK, Eisenberg JF (1989). Brain size and its relation to the rate of metabolism in mammals. *The American Naturalist*.

[89] Palmqvist P, Jiménez-Arenas JM, Pérez-Claros JA (2012). Postcanine tooth size and metabolic requirements in primates. *American Journal of Physical Anthropology*.

[90] Jolly CJ (1970). The seed-eaters: a new model of hominid differentiation based on a baboon analogy. *Man*.

[91] Navarrete A, van Schaik CP, Isler K (2011). Energetics and the evolution of human brain size. *Nature*.

[92] Demes B, Creel N (1988). Bite force, diet, and cranial morphology of fossil hominids. *Journal of Human Evolution*.

[93] Stedman HH, Kozyak BW, Nelson A (2004). Myosin gene mutation correlates with anatomical changes in the human lineage. *Nature*.

[94] Perry GH, Verrelli BC, Stone AC (2005). Comparative analyses reveal a complex history of molecular evolution for human MYH16. *Molecular Biology and Evolution*.

[95] McCollum MA, Sherwood CC, Vinyard CJ, Lovejoy CO, Schachat F (2006). Of muscle-bound crania and human brain evolution: the story behind the MYH16 headlines. *Journal of Human Evolution*.

[96] Charrier C, Joshi K, Coutinho-Budd J (2012). Inhibition of SRGAP2 function by its human-specific paralogs induces neoteny during spine maturation. *Cell*.

[97] Ungar PS, Grine FE, Teaford MF (2008). Dental microwear and diet of the Plio-Pleistocene hominin *Paranthropus boisei*. *PLoS ONE*.

[98] Ferring R, Oms O, Agustí J (2011). Earliest human occupations at Dmanisi (Georgian Caucasus) dated to 1.85-1.78 Ma. *Proceedings of the National Academy of Sciences of the United States of America*.

[99] Lordkipanidze D, Jashashvili T, Vekua A (2007). Postcranial evidence from early *Homo* from Dmanisi, Georgia. *Nature*.

[100] Martinón-Torres M, Bermúdez de Castro JM, Gómez-Robles A (2008). Dental remains from Dmanisi (Republic of Georgia): morphological analysis and comparative study. *Journal of Human Evolution*.

