# Supplementary material for: On the Relationships of Postcanine Tooth Size with Dietary Quality and Brain Volume in Primates: Implications for Hominin Evolution
Source: Biomed Res Int. 2014 Jan 30;2014:406507. doi: 10.1155/2014/406507 (PMC3925621; doi:10.1155/2014/406507)

Supplementary information

Table S1

Table S1. Non-human primate species and variables used in this study. BM: body mass; BrM: brain mass; PCTA: postcanine tooth area; DQ: dietary quality; *: values for *Pigathrix* sp. DQ = 1α + 2β + 3.5γ (α: percentage of leaves and structural parts of plants; β: percentage of fruit and reproductive parts of plants, including nuts and seeds; γ: percentage of animal items).

Species	BM [kg]	ECV [ml]	PCTA [mm^2]	DQ
Galago senegalensis	0.19 [1]	3.96 [1]	40.22 [3]	323 [6]
Lemur catta	2.21 [1]	22.90 [1]	79.04 [4]	158.5 [7]
Lepilemur mustelinus	0.77 [1]	9.56 [1]	30.82 [3]	110 [6]
Nycticebus coucang	0.65 [1]	10.13 [1]	84.95 [3]	164.5 [8]
Otolemur crassicaudatus	1.15 [1]	11.78 [1]	65.19 [3]	205 [6]
Perodicticus potto	0.84 [1]	12.42 [1]	83.84 [3]	223 [6]
Propithecus verreauxi	2.96 [1]	26.21 [1]	114.33 [3]	144 [7]
***Tarsius* (genus)**	0.13 [1]	3.16 [1]	61.85 [3]	350 [6]
Alouatta caraya	5.38 [1]	52.63 [1]	125.29 [3]	130 [9]
Alouatta palliata	6.25 [1]	49.88 [1]	145.83 [3]	136.8 [10]
Alouatta seniculus	5.95 [1]	55.22 [1]	155.69 [3]	134.6 [11]
Aotus trivirgatus	0.99 [1]	16.85 [1]	44.71 [3]	177 [6]
Ateles geoffroyi	7.57 [1]	105.09 [1]	76.82 [3]	181.6 [12]
Brachyteles arachnoides	9.51 [2]	120.10 [2]	138.96 [3]	154.1 [13]
Cacajao calvus	3.17 [1]	76.00 [1]	61.51 [3]	201.6 [14]
Callicebus moloch	0.90 [2]	19.00 [2]	37.45 [3]	187 [6]
Callithrix jacchus	0.32 [1]	7.24 [1]	15.72 [5]	190 [15]
Callithrix pygmaea	0.12 [1]	4.17 [1]	10.05 [5]	183 [16]
Cebus apella	2.94 [1]	66.63 [1]	74.14 [3]	192.2 [17]
Chiropotes satanas	2.64 [2]	53.00 [2]	41.51 [3]	189.3 [18-19]
Lagothrix lagotricha	7.15 [1]	96.50 [1]	98.77 [3]	182.9 [20]
Leontopithecus rosalia	0.61 [1]	12.83 [1]	22.90 [5]	212.5 [21]
Pithecia pithecia	1.76 [1]	32.26 [1]	43.45 [3]	184 [6]
Saguinus oedipus	0.43 [1]	9.76 [1]	20.43 [5]	251 [6]
Saimiri sciureus	0.80 [1]	24.14 [1]	27.45 [3]	266 [22]
Cercocebus galeritus	7.44 [1]	99.07 [1]	163.33 [3]	179.8 [23]
Cercocebus torquatus	8.93 [1]	105.99 [1]	163.77 [3]	175.8 [24]
Cercopithecus ascanius	3.71 [1]	59.58 [1]	68.91 [3]	226.5 [25]
Cercopithecus cephus	3.59 [1]	65.26 [1]	79.85 [3]	247.5 [26]
Cercopithecus mitis	6.11 [1]	71.33 [1]	94.59 [3]	202.8 [25]
Cercopithecus mona	3.72 [1]	61.84 [1]	79.24 [3]	205 [6]
Cercopithecus neglectus	5.45 [1]	65.97 [1]	96.13 [3]	198 [6]
Cercopithecus nictitans	5.41 [2]	78.60 [2]	81.77 [3]	182.9 [27]
Chlorocebus aethiops	3.72 [1]	65.00 [1]	88.61 [3]	196 [28]
Colobus polykomos	9.10 [1]	73.83 [1]	129.59 [3]	134.6 [29]
Lophocebus albigena	7.36*	99.10 [2]	121.90 [3]	233.5 [30]
Macaca fascicularis	4.25 [1]	63.98 [1]	119.30 [3]	185.9 [31]
Macaca mulatta	6.79 [1]	88.98 [1]	140.58 [3]	138 [6]

Macaca nemestrina	8.82 [1]	105.59 [1]	157.72 [3]	195 [6]
Macaca nigra	7.68 [1]	94.90 [1]	131.64 [3]	245.85 [32]
Macaca sylvanus	12.01 [1]	93.20 [1]	98.11 [3]	129 [33]
Nasalis larvatus	14.56 [1]	92.30 [1]	127.07 [3]	145 [34]
Papio cynocephalus	17.15 [1]	163.19 [1]	307.05 [3]	163 [6]
Pliocolobus badius	8.29 [1]	63.59 [1]	118.19 [3]	144.5 [35]
Presbitys comata	6.70 [1]	80.30 [1]	82.57 [3]	125 [36]
Pygathrix nemaeus	9.72 [1]*	97.20 [1]*	123.39 [3]	118 [37]
Rhinopithecus roxellana	12.25 [2]	121.70 [2]	176.36 [3]	160 [38]
Theropithecus gelada	15.35 [1]	133.33 [1]	244.24 [3]	117 [6]
Trachypithecus cristatus	6.39 [1]	57.86 [1]	98.08 [3]	132 [6]
Trachypithecus johnii	11.60 [1]	84.60 [1]	115.88 [3]	132 [6]
Gorilla gorilla	120.10 [1]	490.41 [1]	669.94 [3]	173.2 [27]
Hylobates lar	5.60 [1]	101.97 [1]	87.60 [3]	232 [39]
Hylobates moloch	5.82 [2]	113.70 [2]	100.64 [3]	175 [40]
Pan paniscus	39.10 [1]	341.29 [1]	221.50 [3]	164 [41]
Pan troglodytes	44.97 [1]	368.35 [1]	306.73 [3]	194.6 [27]
Pongo pygmaeus	58.54 [1]	377.38 [1]	434.34 [3]	178 [6]

Table S2

Table S2. Descriptive statistics for the extinct hominin species analyzed in this study ($\overline{x}$: mean; N: sample size, in brackets). BM: body mass; BrM: brain mass; PCTA: postcanine tooth area; LP: Lower Pleistocene; MP: Middle Pleistocene. (*): MD values for P^4, M^1 and M^2 were measured on a photograph.

	BM [kg]	BrM [g]	PCTA [mm^2]
A. afarensis	38 (–) [42]	457.8 (4) [48]	499.0 (P^4=11, M^1=6, M^2=8) [56]
A. africanus	35.5 (–) [42]	448.1 (7) [49]	527.2 (P^4=15, M^1=24, M^2=23) [57]
A. garhi	–	445.8 (1) [50]	674.9 (P^4=1, M^1=1, M^2=1) [50]
P. boisei	41.5 (–) [42]	476.2 (7) [51]	712.6 (P^4=6, M^1=10, M^2=8) [58, 59]
P. robustus	36 (–)[42]	470.9 (1) [52]	569.2 (P^4=22, M^1=26, M^2=23) [57]
A. sediba	33 (1) [43]	437.1 (1) [43]	442.9 (P^4=1, M^1=1, M^2=1) [43]
H. habilis	34.5 (–) [42]	610.8 (5) [51]	457.4 (P^4=9, M^1=13, M^2=8) [58, 60]
H. rudolfensis	55.5 (–) [42]	735.8 (1) [51]	613.3 (P^4=1, M^1=1, M^2=1) [58]
H. georgicus	43.4 (3) [44]	654.5 (4) [53-55]	405.3 (P^4=2, M^1=2, M^2=2) [53, 61]
H. ergaster	61.7 (5) [45]	860.6 (4) [45]	434.2 (P^4=2, M^1=3, M^2=4) [58, 62]
H. erectus (LP)	–	842.6 (7) [45]	420.8 (P^4=4, M^1=3, M^2=3) [62]
H. erectus (MP)	56.2 (2) [45]	1082.5 (17) [45]	365 (P^4=11, M^1=7, M^2=8) [62]
H. heidelbergensis	62 (–) [45]	1161.7 (9) [45]	377.8 (P^4=8, M^1=9, M^2=9) [62]
H. rhodesiensis	65.8 (7) [45]	1053.3 (5) [45]	410.3 (P^4=1, M^1=1, M^2=1) [62]
H. neanderthalensis	74.4 (21) [45]	1414.3 (16) [45]	331.1 (P^4=20, M^1=20, M^2=19) [62]
H. floresiensis	36 (1) [46]	413.8 (1) [46]	347.1 (P^4=1, M^1=1, M^2=1) [46] (*)
H. sapiens	65.8 (81) [47]	1462.7 (37) [45]	320.2 (P^4=27, M^1=38, M^2=28) [47, 63]

Table S3

Table S3. Descriptive statistics of regression analyses of brain mass (BrM, in g) on body mass (BM, in kg) and of body mass on postcanine tooth area (PCTA, in mm^2) for primate groups. RMA: Reduced Major Axis; OLS: Ordinary Least Squares; N: sample size; r: coefficient of correlation; β_0: Y–intercept; β_1: slope; [CI 95%]: 95% confidence interval for the slope; H_0 (b=0): null hypothesis for slope zero; H_0 (b=1; b=2/3): null hypothesis for slope values expected from geometric scaling (isometry); H_0 (b=3/4) and H_0 (b=1/2): null hypothesis for slope values expected from elastic similarity (allometry); n.s.: non-significant t-test value for comparison of slopes (p > 0.05).

**logBrM-logBM**		**N**	**r**	**β_0**	**β_1 [CI 95%]**	**$H_0(\beta_1=0)$**	**$H_0(\beta_1=1)$**	**$H_0(\beta_1=3/4)$**
All primates	RMA	71	0.973	1.220	0.902 [0.842-0.956]	<0.0001	<0.0001	<0.0001
	OLS	71	0.973	1.240	0.877 [0.815-0.936]	<0.0001	<0.0001	<0.0001
Non-human primates	RMA	56	0.972	1.257	0.787 [0.739-0.829]	<0.0001	<0.0001	n.s.
	OLS	56	0.972	1.207	0.765 [0.719-0.808]	<0.0001	<0.0001	n.s.
Prosimians	RMA	8	0.993	1.114	0.687 [0.634-0.734]	<0.0001	<0.0001	<0.05
	OLS	8	0.993	1.113	0.682 [0.642-0.743]	<0.0001	<0.0001	<0.05
Anthropoids	RMA	48	0.97	1.322	0.723 [0.691-0.774]	<0.0001	<0.0001	n.s.
	OLS	48	0.97	1.338	0.705 [0.691-0.774]	<0.0001	<0.0001	n.s.
Platyrrhines	RMA	17	0.967	1.317	0.774 [0.692-0.855]	<0.0001	<0.0005	n.s.
	OLS	17	0.967	1.323	0.748 [0.668;0.837]	<0.0001	<0.0001	n.s.
Catarrhines	RMA	31	0.936	1.361	0.684 [0.599-0.763]	<0.0001	<0.0001	n.s.
	OLS	31	0.936	1.404	0.640 [0.563-0.751]	<0.0001	<0.0001	n.s.
Cercopithecoids	RMA	25	0.656	1.433	0.581 [0.440-0.706]	<0.0001	<0.0001	<0.005
	OLS	25	0.656	1.529	0.470 [0.342-0.609]	<0.0001	<0.0001	<0.005
Hominoids	RMA	6	0.991	1.643	0.540 [0.471-0.781]	<0.0002	<0.0005	n.s.
	OLS	6	0.991	1.65	0.535 [0.459-0.768]	<0.0002	<0.0005	n.s.
Extinct hominins	RMA	15	0.938	0.19	1.581 [1.321-1.847]	<0.0001	<0.0001	<0.0001
	OLS	15	0.938	0.356	1.489 [1.205-1.786]	<0.0001	<0.0001	<0.0001
Homo	RMA	10	0.9	0.317	1.519 [0.854-1.940]	<0.0001	n.s.	<0.05
	OLS	10	0.900	0.579	1.367 [0.828-1.906]	<0.0005	n.s.	<0.05
**logBM-logPCTA**		**N**	**r**	**β_0**	**β_1 [CI 95%]**	**$H_0(\beta_1=0)$**	**$H_0(\beta_1=2/3)$**	**$H_0(\beta_1=1/2)$**
All primates	RMA	71	0.917	1.620	0.583 [0.518-0.650]	<0.0001	<0.05	<0.0001
	OLS	71	0.917	1.660	0.534 [0.448-0.616]	<0.0001	<0.05	<0.0001
Non-human primates	RMA	56	0.883	1.633	0.539 [0.461-0.627]	<0.0001	<0.01	n.s.
	OLS	56	0.883	1.671	0.475 [0.369-0.585]	<0.0001	<0.01	n.s.
Prosimians	RMA	8	-	-	-	n.s.	-	-
	OLS	8	-	-	-	n.s.	-	-
Anthropoids	RMA	48	0.961	1.526	0.627 [0.580-0.663]	<0.0001	<0.05	<0.001
	OLS	48	0.961	1.554	0.602 [0.561-0.643]	<0.0001	<0.05	<0.005
Platyrrhines	RMA	17	0.959	1.531	0.643 [0.549;0.720]	<0.0001	n.s.	<0.0005
	OLS	17	0.959	1.538	0.616 [0.529;0.698]	<0.0001	n.s.	<0.0001
Catarrhines	RMA	31	0.912	1.515	0.633 [0.506-0.711]	<0.0001	n.s.	<0.05
	OLS	31	0.912	1.570	0.577 [0.500-0.689]	<0.0001	n.s.	<0.05
Cercopithecoids	RMA	25	0.764	1.404	0.774 [0.591-0.973]	<0.0001	n.s.	n.s.
	OLS	25	0.764	1.563	0.592 [0.372-0.837]	<0.0001	n.s.	n.s.
Hominoids	RMA	6	0.980	1.464	0.635 [0.267-0.774]	<0.001	n.s.	n.s.
	OLS	6	0.980	1.482	0.622 [0.264-0.758]	<0.001	n.s.	n.s.
Extinct hominins	RMA	15	-0.471	4.104	-0.817 [-1.175 — -0.463]	n.s.	n.s.	-
	OLS	15	-0.471	3.290	-0.385 [-0.759 — -0.078]	n.s.	n.s.	-
Homo	RMA	10	-	-	-	n.s.	-	-
	OLS	10	-	-	-	n.s.	-	-

Table S4

Table S4. Descriptive statistics of regression analyses of postcanine tooth area (PCTA) on brain mass (BrM) in several primate groups. For legend see Table S3; res: size-adjusted residuals from the equations for logPCTA and logBrM on logBM.

**logBrM-logPCTA**		**N**	**r**	**β_0**	**β_1 [CI 95%]**	**H_0 (β_1=0)**	**H_0 (β_1=isom)**
All primates	RMA	73	0.884	0.831	0.646 [0.555-0.734]	<0.0001	*n.s.*
	OLS	73	0.884	0.978	0.571 [0.477-0.666]	<0.0001	<0.05
Non-human primates	RMA	56	0.668	0.773	0.684 [0.564-0.809]	<0.0001	n.s.
	OLS	56	0.668	0.989	0.559 [0.408-0.715]	<0.0001	n.s.
Prosimians	RMA	8	-	-	-	ns	-
	OLS	8	-	-	-	ns	-
Anthropoids	RMA	48	0.927	-0.448	1.160 [1.056-1.277]	<0.0001	<0.0001
	OLS	48	0.927	-0.280	1.075 [0.935-1.123]	<0.0001	<0.0001
Platyrrhines	RMA	17	0.882	0.437	0.831 [0.635-0.974]	<0.0001	n.s.
	OLS	17	0.882	0.587	0.732 [0.565-0.878]	<0.0001	n.s.
Catarrhines	RMA	31	0.894	0.255	0.926 [0.710-1.093]	<0.0001	<0.001
	OLS	31	0.894	0.454	0.828 [0.631-1.031]	<0.0001	*n.s.*
Cercopithecoids	RMA	25	0.869	-0.505	1.333 [1.143-1.548]	<0.0001	<0.0001
	OLS	25	0.869	-0.167	1.158 [0.929-1.521]	<0.0001	<0.0001
Hominoids	RMA	6	0.951	-0.465	1.174 [0.903-1.528]	<0.005	n.s.
	OLS	6	0.951	-0.327	1.117 [0.757-1.407]	<0.005	n.s.
Extinct hominins	RMA	15	-0.645	4.211	-0.547 [-0.713 – -0.387]	<0.001	n.s.
	OLS	15	-0.645	3.658	-0.353 [-0.568 – -0.157]	<0.002	n.s.
Homo	RMA	11	-	-	-	n.s.	-
	OLS	11	-	-	-	n.s.	-
Homo without *H. floresiensis*	RMA	10	-0.751	4.407	-0.604 [-0.897– -0.247]	<0.02	ns
	OLS	10	-0.751	3.96	-0.436[-0.695;-0.083]	<0.02	ns
**$logPCTA_{BMres}$ on $logBrM_{BMres}$**		**N**	**r**	**β_0**	**β_1[CI 95%]**	**H_0(β_1=0)**	**H_0(β_1=isom)**
All Primates	OLS	71	-	-	-	n.s.	-
Non-human Primates	OLS	56	-	-	-	n.s.	-
Prosimii	OLS	8	0.811	0.407	1.791 [0.489-3.084]	<0.05	n.s.
Anthropoids	OLS	48	-0.301	-0.073	-0.227 [-0.441 – -0.013]	<0.05	<0.005
Platyrrhines	OLS	17	-0.697	-0.071	-0.588 [-0.922 – -0.225]	<0.005	<0.05
Catarrhines	OLS	31	-	-	-	n.s.	-
Cercopithecoids	OLS	25	-	-	-	n.s.	-
Hominoids	OLS	6	-	-	-	n.s.	-
Extinct hominins	OLS	15	-0.810	0.252	-1.146 [-1.646 – -0.651]	<0.005	n.s.
Homo	OLS	10	-0.650	0.162	-0.779 [-1.521 – -0.037]	<0.05	n.s.
**$logPCTA_{DQres}$ on $logBrM_{DQres}$**		**N**	**r**	**β_0**	**β_1[CI 95%]**	**H_0(β_1=0)**	**H_0(β_1=isom)**
Non-human Primates	OLS	56	0.791	0.000	0.543 [0.429-0.658]	<0.0001	<0.05
Prosimians	OLS	8	0.713	0.441	0.838 [0.016-1.660]	<0.05	n.s.
Anthropoids	OLS	48	0.902	-0.063	0.736 [0.632-0.841]	<0.0001	n.s.
Platyrrhines	OLS	17	0.824	-0.124	0.625 [0.389-0.861]	<0.0001	n.s.
Catarrhines	OLS	31	0.882	-0.037	0.724 [0.576-0.871]	<0.0001	n.s.
Cercopithecoids	OLS	25	0.813	-0.029	0.741 [0.511-0.970]	<0.0001	n.s.
Hominoids	OLS	6	0.914	-0.382	1.154 [0.443-1.866]	<0.02	n.s.

Table S5

Table S5. Descriptive statistics of regression analyses of dietary quality (DQ) postcanine tooth area (PCTA, in mm^2) in several primate groups. Res: size-adjusted residuals from the equations for logPCTA and logDQ on logBM and logBrM respectively.

**logDQ-logPCTA**		**N**	**r**	**β_0**	**β_1 [CI 95%]**	**$H_0(\beta_1=0)$**
Non-human primates	RMA	56	-0.35	2.871	-0.318[-0.401 – -0,190]	<0.01
	OLS	56	-0.35	2.467	-0.112[-0.179 – -0.013]	<0.01
Prosimians	RMA	8	-	-	-	n.s.
	OLS	8	-	-	-	n.s.
Anthropoids	RMA	48	-0.41	2.764	-0.264[-0.331 – -0.158]	<0.005
	OLS	48	-0.41	2.456	-0.108[-0.158 – -0.035]	<0.005
Platyrrhines	RMA	17	-0.70	2.659	-0.234[-0.322 – -0.134]	<0.002
	OLS	17	-0.70	2.538	-0.163[-0.258 – -0.035]	<0.002
Catarrhines	RMA	31	-	-	-	n.s.
	OLS	31	-	-	-	n.s.
Cercopithecoids	RMA	25	-	-	-	n.s.
	OLS	25	-	-	-	n.s.
Hominoids	RMA	6	-	-	-	n.s.
	OLS	6	-	-	-	n.s.
**$logDQ_{BMres}$ on $logPCTA_{BMres}$**		**N**	**r**	**β_0**	**β_1[CI 95%]**	**$H_0(\beta_1=0)$**
Non-human primates	OLS	56	-	-	-	n.s.
Prosimians	OLS	8	0.797	-0.14	0.526[0.128-0.924]	<0.02
Anthropoids	OLS	48	-	-	-	n.s.
Platyrrhines	OLS	17	-0.60	-0.05	-0.369[-0.642– -0.096]	<0.02
Catarrhines	OLS	31	-	-	-	n.s.
Cercopithecoids	OLS	25	-	-	-	n.s.
Hominoids	OLS	6	-	-	-	n.s.
**$logDQ_{BrMres}$ on $logPCTA_{BrMres}$**		**N**	**r^2**	**β_0**	**β_1[CI 95%]**	**$H_0(\beta_1=0)$**
Non-human primates	OLS	56	-	-	-	n.s.
Prosimians	OLS	8	0.592	-0.19	0.700[0.235-1.165]	<0.05
Anthropoids	OLS	48	0.109	-0.01	-0.186[-0.339– -0.033]	<0.05
Platyrrhines	OLS	17	0.549	-0.05	-0.314[-0.457– -0.171]	<0.001
Catarrhines	OLS	31	-	-	-	n.s.
Cercopithecoids	OLS	25	-	-	-	n.s.
Hominoids	OLS	6	-	-	-	n.s.

Table S6

Table S6. Descriptive statistics of the regressions adjusted for Primates after independent contrasts analyses using the Phylogenetic Generalized Least-Squares (PGLS) method. α: estimated parameter used to indicate the strength of the evolutionary constraint.

**Primates**		**N**	**α**	**r**	**β_0**	**β_1 [CI 95%]**	**se**	**H_0 (β_1=0)**	**H_0 (β_1=isom)**	**H_0 (β_1=0.75)**
logBrM on logBM	PGLS	56	0.95	0.94	1.21	0.69 [0.62-0.76]	0.035	<0.0001	<0.0001	n.s.
logPCTA on logBM	PGLS	56	-	-	-	-	-	n.s.	-	-
logPCTA on logBrM	PGLS	56	0.50	0.78	0.98	0.69 [0.54-0.84]	0.076	<0.0001	<0.0001	-
logDQ on logBM	PGLS	56	3.51	-0.44	2.30	-0.10 [-0.24 – -0.64]	0.028	<0.05	-	-
logDQ on logBrM	PGLS	56	-	-	-	-	-	n.s.	-	-
logDQ on logPCTA	PGLS	56	2.63	-0.28	2.50	-0.12 [-0.04 – -0.62]	0.057	<0.05	-	-

**Primates**		**N**	**α**	**r**	**β_0**	**β_1**	**se**	**H_0 (β_1=0)**	**H_0 (β_1=isom)**	**H_0 (β_1=0.75)**
$logPCTA_{BMres}$ on $logBrM_{BMres}$	PGLS	56	-	-	-	-	-	n.s.	-	-
$logDQ_{BMres}$ on $logBrM_{BMres}$	PGLS	56	-	-	-	-	-	n.s.	-	-
$logDQ_{BMres}$ on $logPCTA_{BMres}$	PGLS	56	-	-	-	-	-	n.s.	-	-
$logDQ_{BrMres}$ on $logPCTA_{BrMres}$	PGLS	56	-	-	-	-	-	n.s.	-	-

Body and brain size

BM, BrM and ECV values for extant primates and fossil hominins were taken from the bibliography (Tables S1 and S2). For those species in which brain size was expressed in the literature as endocranial volumes (ECV, in cm^3), BrM was calculated using the following formula:

$$BrM = 1.036\ ECV\ [1].$$

The sample analyzed of *H. sapiens* includes only Late Pleistocene specimens from Europe and the Near East (see Table S2).

Postcanine tooth size

Following previous studies, PCTA was estimated by summing the occlusal areas of three maxillary teeth, the fourth premolar (P^4) and the first and second molars (M^1 and M^2). The area of each tooth was obtained multiplying its mesiodistal length (MD) by its buccolingual breadth (BL). Most measurements were collected from the seminal work of Swindler [3] on the dentition of primates. The database was completed with metric data of *Lemur catta* [4] and Callitrichinae [5]. The measurements for fossil hominin crania were taken from several bibliographic sources (Table S2). The only exception was holotype LB-1 of *Homo floresiensis,* whose MD values for P^4, M^1 and M^2 were measured on a photograph [ref. 46: Fig. 1]. It is worth making clear that after measuring the buccolingual dimensions of selected teeth for fossil *Homo* on published photographs [64, 65], the differences with the measurements obtained from the bibliography were of <0.1 mm in all cases.

It could be argued, however, that the use of only three teeth of the postcanine dentition could introduce a source of error and bias in our analyses, except for those constructed within a narrow taxonomy. For example, if M^1:M^3 size ratio is compared between platyrrhines and cercopithecoids, it is apparent that New World monkeys devote relatively little area to the M^3 in comparison to Old World monkeys. This is particularly evident in callitrichids, which mostly lack an M^3, accounting the M^1 and M^2 for 100% of the molar area. In contrast, the M^3 represents a substantial proportion of total molar area in the catarrhines, reaching >33% in some species. This is a potential source of fluctuation for the slopes adjusted when different primate clades are involved (i.e., the slopes would not only reflect differences in tooth size relative to body size, but also tooth sizes relative to teeth not included in the calculation of postcanine tooth area). However, the reason for analyzing exclusively the area of P^4, M^1 and M^2 is that McHenry [66] used these teeth for calculating the molarization coefficient and measurements for a high number of primate species are available. In contrast, the use of other postcanine teeth would have resulted in a substantial decrease of sample size. In addition, the first molar is usually placed at 7:10 of the jaw lever arm, the point where the maximum bite force is exerted during chewing, while the fourth premolar and the second molar are situated immediately before and after it, respectively, which means that these teeth are also crucial for mastication [66]. Finally, we are aware that teeth are complex structures and the use of postcanine tooth area oversimplifies a number of histological, morphological and topological aspects that play a key role in food processing. However, there is a vast number of meta-analyses in which this type of variables (i.e., body size, brain size and tooth size) have been used (e.g., for the specific case of postcanine tooth area [67].

Dietary Quality

Dietary quality (DQ) was defined as follows:

$$DQ = 1\alpha + 2\beta + 3.5\gamma\ [68],$$

where α is the percentage of leaves and structural parts of plants in the diet, β is the percentage of fruit and reproductive parts of plants (including nuts and seeds), and γ is the percentage of animal items. In this way, DQ reflects the dietary contribution of those foodstuffs considered in more traditional dietary categories (i.e., folivores, frugivores and faunivores). DQ was estimated for primate species using data on diet composition published in a number of studies (see Table S1). This variable measures some physical properties of foodstuffs, for example those derived from fiber contents, but others (e.g., toughness and hardness) are not clearly reflected in DQ values. However, DQ has been widely used in this type of approaches, including some recent studies [e.g., 69, 70].

In addition, we must recognize that our analyses may oversimplify the relationship between tooth size and food type by focusing exclusively on dietary quality. The reason is that enamel thickness and tooth morphology and topology may reflect a balance between the need to fracture foods without fracturing the tooth itself: for example, the large teeth of *Paranthropus* could have been used for processing foodstuffs with quite divergent mechanical properties, which opens the possibility that tooth morphology reflects "fallback" food items rarely consumed instead of the most commonly masticated items [71-74].

Phylogenetic Control

The procedure used of Phylogenetic Generalized Least-Squares (PGLS) works as follows [75]: imagine eight species showing the phylogenetic relationship depicted in Figure S2. Two phenotypic traits (X, Y; for example, body mass and brain volume) has been measured in each of these species for analyzing the relationship between the traits. The problem is that it is not possible to assume that the measurements taken in any species will be independent from those obtained in others, as they may be subject to phylogenetic inertia.

The method of phylogenetic contrasts consists of transforming the original metric variables into new variables (CX, CY), which preserve the same covariance as the original ones. According to the tree topology of the hypothetical phylogeny analyzed (Fig. S1), the differences X1-X2, X3-X4, X5-X6, and X7-X8 will be independent of each other. For example, the difference X1-X2 will depend exclusively on the evolutionary events that took place in branches 1 and 2, while the difference X3-X4 will be the result of those that account for branches 3 and 4. Given that both sets of events are independent, this situation guarantees data independence for statistical analyses.

According to this reasoning and deepening into the tree nodes, we can affirm that the difference [(X1+X2)/2] – [(X3+X4)/2] is independent from the difference [(X5+X6)/2] – [(X7+X8)/2] and thus, the original variable X (with N species) is finally transformed in CX (with N-1 cases, dots in Fig. 1). The same procedure is applied to the variable Y for transforming it in CY. Given that the method ensures that cov(X,Y) = cov(CX,CY), this implies that if the original variables were correlated the new contrasts will show also a statistical correlation [for technical details, see ref. 76], but now it is guaranteed that the cases studied are independent (i.e., free of phylogenetic inertia).

COMPARE calculates the relationship between traits, while also taking phylogeny and within-taxon variation into account. As with estimation of ancestral states, weights are a function of the within-species variation, the phylogeny, and the model of character evolution.

Among other things, the PGLS method implemented in COMPARE assumes that:

1. - Character evolution can be described using a model of phenotypic evolution, which leads to either a linear or an exponential increase in between-taxon divergence with phylogenetic distance. Exponential models are appropriate for evolution under some

constraints (e.g., stabilizing selection about a fixed optimum). A linear model (e.g., Brownian motion) is approximated by setting the constraint to zero. This is more commonly used in population genetics for describing the evolution of characters undergoing random genetic drift, or directional selection when the direction of selection is shifting back and forth at random.

2. - Expected similarities and differences between all taxa (including hypothetical ancestors) are known. These similarities or differences will be obtained directly from the phylogeny as part of the calculations.

3. - Standard errors of the measurements for each taxon are available, and they adequately represent within-species variation in the trait.

4. - Relationships between traits are well described by a generalized least-squares model regression or correlation.

References

[1] Isler K, Kirk EC, Miller JMA, Albrecht GA, Gelvin BR et al. (2008) Endocranial volumes of primate species: scaling analyses using a comprehensive and reliable data set. J Hum Evol 55: 967–978.

[2] Barton R (1999) The evolutionary ecology of the primate brain. In: Lee PC, editor. Comparative Primate Socioecology. Cambridge: Cambridge University Press. pp. 167–194.

[3] Swindler DR (1976) Primate dentition: An introduction to the teeth of non–human primates. Cambridge: Cambridge University Press. 312 p.

[4] Sauther ML, Cuozzo FP, Sussman RW (2001) Analysis of dentition of a living wild population of ring–tailed lemurs (Lemur catta) from Beza Mahafaly, Madagascar. Am J Phys. Anthropol 114: 215–223.

[5] Plavcan JM, Gómez AM (1993) Dental scaling in the callitrichinae. Int J Primatol 14: 177–192.

[6] Sailer LD, Gaulin SJC, Boster JS, Kurland JA (1985) Measuring the relationship between dietary quality and body size in primates. Primates 26: 14–27.

[7] Simmen B, Hladick A, Ramasiarisoa P (2003) Food intake and dietary overlap in native *Lemur catta* and *Propithecus verreauxi* and introduced *Eulemur fulvus* at Berenty, Southern Madagascar. Int J Primatol 24: 949-968.

[8] Wiens F (2002) Behavior and ecology of wild slow lorises (Nycticebus coucang): social organization, infant care system, and diet. Unpublished Ph. D. dissertation, Bayreuth University, Bayreuth, 118 p.

[9] Bravo, SP, Sallenave A (2003) Foraging behavior and activity patterns of *Alouatta caraya* in the Northeastern Argentinean flooded forest. Int J Primatol 24: 825-846.

[10] Bal-Azkarate JC, Arroyo-Rodriguez V (2007) Diet and activity pattern of howler monkeys (*Alouatta palliata*) in Los Tuxtlas, Mexico: Effects of habitat fragmentation and implications for conservation. Am J Primatol 69: 1013-1029.

[11] Julliot C, Sabatier D (1993) Diet of the red howler monkey (*Alouatta seniculus*) in French Guiana. Int J Primatol 14: 527-550.

[12] González-Zamora A, Arroyo-Rodríguez V, Chaves OM, Sánchez-López S, Stoner KE, Riba-Hernández P (2009) Diet of spider monkeys (*Ateles geoffroyi*) in Mesomerica: Current knowledge and future directions. Am J Primatol 71: 8-20.

[13] De Carvalho O Jr, Ferrari SF, Strier KB (2004) Diet of muriqui (*Brachyteles arachnoides*) in continuous primary forest. Primates 45: 201-204.

[14] Bowler M, Bodmer RE (2011) Diet and Food Choice in Peruvian Red Uakaris (Cacajao calvus ucayalii): Selective or Opportunistic Seed Predation? Int J Primatol 32: 1109-1122.

[15] Soares de Castro CS, Araújo R (2007) Diet and feeding behavior of marmoset, *Callithrix jacchus.* Braz. J. Ecol. 7: 14-19.

[16] Townsend WR (2001) *Callithrix pygmaea*. Mammalian Species 665: 1-6.

[17] Galettia M, Pedronia F (1994) Seasonal diet of capuchin monkeys (*Cebus apella*) in a semideciduous forest in south-east Brazil. J. Trop. Ecol. 10: 27-39.

[18] Port-Carvalho M, Ferrari SF (2004) Occurrence and diet of the black beared saki (*Chiropotes satanas satanas*) in the fragmented landscape of western Maranhao, Brazil. Neotrop. Primates 12: 17-21.

[19] Veiga LM, Ferrari SF (1006) Predation of arthropods by southern beared sakis (*Chiropotes satanas*) in Eastern Brazilian Amazonia. Am. J. Primatol. 68: 209-215.

[20] Defler TR, Defler SB (1996) Diet of a group of *Lagothrix lagothricha lagothricha* in southeastern Colombia. Int. J. Primatol. 17:161–190.

[21] Dietz JM, Peres CA, Pinder L (1997) Foraging Ecology and Use of Space in Wild Golden Lion Tamarins (*Leontopithecus rosalia*). Am. J. Primatol. 41: 289-305.

[22] Lima EM, Ferrari SF (2003) Diet of a free-ranging group of squirrel monkeys (*Saimiri sciureus*) in Eastern Brazilian Amazonia. Folia Primatol. 74:150–158.

[23] Kinnaird M (1990) Pregnancy, gestation, and parturition in free-ranging Tana River crested mangabeys (*Cercocebus galeritus galeritus*). Am. J. Primatol. 22: 285-289.

[24] Mitani M (1989) *Cercocebus torquatus*: Adaptive feeding and ranging behaviors related to seasonal fluctuactions of food resources in the Tropical Rain Forest of South-western Cameroon. Primates 30: 307-323.

[25] Cords M (1986) Interspecific and intraspecific variation in diet of two forest guenons, Cercopithecus ascanius and C. Mitis. J. Anim. Ecol. 55: 811-827.

[26] Tutin CEG (1999) Fragmented living: Behavioural ecology of Primates in a forest fragmented in the Lopé Reserve, Gabon. Primates 40: 249-265.

[27] Tutin CEG, Ham RM, White LJT, Harrison MJS (1997) The primate community of the Lopé Reserve, Gabon: Diets, responses to fruit scarcity, and effects on biomass. Am. J. Primatol. 42: 1-24.

[28] Harrison MJS (1983) Age and sex differences in the diet and feeding strategies of the green monkey, *Cercopithecus sabaeus*. Anim. Beh. 31: 969-977.

[29] Dasilva GL (1994) Diet of *Colobus polykomos* on Tiwai Island: Selection of food in relation to its seasonal abundance and nutritional quality. Int. J. Primatol. 15: 655-680.

[30] Poulsen JR, Clark CJ, Smith TB (2001) Seasonal variation in the feeding ecology of the grey- cheeked mangabey (*Lophocebus albigena*) in Cameroon. Am. J. Primatol. 54: 91–105.

[31] Yeager CP (1996) Feeding ecology of the long-tailed macaque (*Macaca fascicularis*) in Kalimantan Tengah, Indonesia. Int. J. Primatol. 17: 51-62.

[32] O'Brien TG, Kinnaird MF (1997) Behavior, diet, and movements of the Sulawesi crested black macaque (*Macaca nigra*). Int. J. Primatol. 18: 321-35.

[33] Ménard N (2002) Ecological plasticity of Barbary macaques (*Macaca sylvanus*). Evol. Anthropol. Suppl. 1: 95-100.

[34] Yaeger CP (1989) Feeding ecology of the proboscis monkey (*Nasalis larvatus*). Int. J. Primatol. 10: 497-530.

[35] Decker BS (1994) Effects of habitat disturbance on the behavioral ecology and demographics of the Tana River red colobus (*Colobus badius rufomitratus*). Inter. J. Primatol 15: 703-737.

[36] Ruhiyat Y (1983) Socio-ecological study of *Presbytis aygula* in West Java. Primates 24: 344-359.

[37] Lippold L K (1977). The douc langur: A time for conservation. In: Prince Rainier HSH, Bourne GH, editors. Primate Conservation. New York: Academic Press, pp. 513–538.

[38] Guo S, Baoguo L, Watanabe K (2007) Diet and activity budget of *Rhinopithecus roxellana* in the Qinling Mountains, China. Primates 48: 268–276.

[39] Palombit RA (1997) Inter- and intraspecific variation in the diets of sympatric siamang (*Hylobates syndactylus*) and Lar gibbons (*Hylobates lar*). Folia Primatologica 68: 321-337.

[40] Sanha K, Lappan S, Choe JC (2011) Diet and ranging behavior of the endangered Javan Gibbon (*Hylobates moloch*) in a submontane tropical rainforest. Am. J. Primatol. 73: 270-280.

[41] Badrian N, Badrian A, Susman R (1981) Preliminary Observations on the Feeding Behavior of Pan paniscus in the Lomako Forest of Central Zaire. Primates 22: 173-181.

[42] McHenry, H.M., Coffing, K., 2000. *Australopithecus* to *Homo:* transformations in body and mind. Annu. Rev. Anthropol. 29: 125–146.

[43] Berger LR, de Ruiter DJ, Churchill SE, Schmid P, Carlson KJ et al. (2010) Australopithecus sediba: a new species of *Homo*-like australopith from South Africa. Science 328: 195–204.

[44] Lordkipanidze D, Jashashvili T, Vekua A, Ponce de León MS, Zollikofer CP, Rightmire GP, Pontzer H, Ferring R, Oms O, Tappen M, Bukhsianidze M, Agusti J, Kahlke R, Kiladze G, Martinez-Navarro B, Mouskhelishvili A, Nioradze M, Rook L (2007) Postcranial evidence from early Homo from Dmanisi, Georgia. Nature 449: 305–310.

[45] Ruff CB, Trinkaus E, Holliday TW (1997) Body mass and encephalization in Pleistocene *Homo*. Nature 387: 173–176.

[46] Brown P, Sutikna T, Morwood MJ, Soejono RP, Jatmiko et al. (2004) A new small-bodied hominin from the Late Pleistocene of Flores, Indonesia. Nature 431: 1055–1061.

[47] Brabant H (1970) La denture humaine an Paléolithique Supérior d'Europe. In: Camps G, Olivier G, editors. L'Homme de Cro–Magnon. Paris: Centre de Recherches Anthropologiques, Préhistoriques et Etnographiques. pp. 99–119.

[48] Holloway RL, Yuan MS (2004) Endocranial morphology of AL 444-2. In: Kimbel WH, Rak Y, Johanson DC, editors. The skull of *Australopithecus afarensis*. New York: Oxford University Press. pp. 123–135.

[49] Neubauer S, Gunz P, Mitteroecker P, Weber GW (2004) Three-dimensional digital imaging of the partial *Australopithecus africanus* endocranium MLD 37/38. Can. Assoc. Radiol. J. 55: 271–278.

[50] Asfaw B, White T, Lovejoy O, Latimer B, Simpson S, Suwa G (1999) *Australopithecus garhi*: a new species of early hominid from Ethiopia. Science 284: 629–635.

[51] Elton S, Bishop LC, Wood B (2001) Comparative context of Plio-Pleistocene hominin brain evolution. J. Hum. Evol. 41: 1–27.

[52] Conroy GC, Falk D, Guyer J, Weber GW, Seidler H et al. (2000) Endocranial capacity in Sts 71 (*Australopithecus africanus*) by three-dimensional computed tomography. Anat. Rec. 258: 391–396.

[53] Gabunia L, Vekua A, Lordkipanidze D, Swisher CC, Ferring R et al. (2000) Earliest Pleistocene hominid cranial remains from Dmanisi, Republic of Georgia: taxonomy, geological setting, and age. Science 288: 1019–1025.

[54] Lordkipanidze D, Vekua A, Ferring R, Rightmire GP, Zollikofer CPE et al. (2006) A fourth hominin skull from Dmanisi, Georgia. Anat. Rec. A Discov. Mol. Cell. Evol. Biol. 288: 1146–1157.

[55] Vekua A, Lordkipanidze D, Rigthtmire GP, Agustí J, Ferring R et al. (2002) A new skull of early *Homo* from Dmanisi, Georgia. Science 297: 85–89.

[56] Kimbel WH, Rak Y, Johanson DC (2004) The skull of *Australopithecus afarensis*. New York: Oxford University Press. p. 272

[57] Moggi–Cecchi J, Grine FE, Tobias PV (2006) Early hominid dental remains from Members 4 and 5 of the Sterkfontein Formation (1966–1996 excavations): catalogue, individual associations, morphological descriptions and initial metrical analysis. J Hum Evol 50: 239–328.

[58] Wood BA (1991) Koobi Fora Research Project. Volume 4: Hominid Cranial Remains. New York: Oxford University Press. 496 p.

[59] Suwa G, Asfaw B, Beyene Y, White TD, Katoh S et al. (1997) The first skull of *Australopithecus boisei*. Nature 389: 489–492.

[60] Kimbel WH, Johanson DC, Rak Y (1997) Systematic assessment of a maxilla of *Homo* from Hadar, Ethiopia. Am. J. Phys. Anthropol. 103: 235–262.

[61] Rightmire GP, Lordkipanidze D, Vekua A (2006) Anatomical descriptions, comparative studies and evolutionary significance of the hominin skulls from Dmanisi, Republic of Georgia. J. Hum. Evol. 50: 115–141.

[62] Bermúdez de Castro JM (1993) The Atapuerca dental remains. New evidence (1987–1991 excavations) and interpretations. J. Hum. Evol. 24: 339–371.

[63] Vandermeersch, B., 1981. Les hommes fosiles de Qafzeh (Israël). Paris: Université Pierre et Marie Curie. p. 319.

[64] Schwartz JH, Tattersall I (2003) The human fossil record, vol. 1: Craniodental morphology of genus *Homo* (Africa and Asia). Hoboken, NJ: John Wiley & Sons. 603 p.

[65] Schwartz JH, Tattersall I (2003) The human fossil record, Vol. 2: Craniodental morphology of genus *Homo* (Africa and Asia). Hoboken, NJ: John Wiley & Sons, 616 p.

[66] Wright BW (2005) Craniodental biomechanics and dietary toughness in the genus *Cebus*. J. Hum. Evol. 48: 473-492.

[67] Copes LE, Schwartz GT (2010) The scale of it all: postcanine tooth size, the taxon-level effect, and the universality of Gould's scaling law. Paleobiology 36:188–203.

[68] Sailer LD, Gaulin SJC, Boster JS, Kurland JA (1985) Measuring the relationship between dietary quality and body size in primates. Primates 26: 14–27.

[69] Snodgrass JJ, Leonard WR, Robertson ML (2009) The energetics of encephalization in early hominids. In: JJ Hublin & M Richards (eds.) Evolution of Hominid Diets: Integrating Approaches to the Study of Palaeolithic Subsistence. Dordrecht: Springer, pp. 15-29.

[70] Allen KL, Kay RF (2011) Dietary quality and encephalization in platyrrhine primates. Proc. R. Soc. B 279: 715-721.

[71] Grine FE, Martin LB (1988) Enamel thickness and development in Australopithecus and Paranthropus. In: Grine FE, editor. Evolutionary History of the ‘‘Robust’’ Australopithecines. New York: Aldine de Gruyter, pp. 3–42.

[72] Laden G, Wrangham R (2005) The rise of the hominids as an adaptive shift in fallback foods: plant underground storage organs (USOs) and australopith origins. J. Hum. Evol. 49: 482–498.

[73] Wood BA, Constantino P (2007) *Paranthropus boisei*: Fifty years of evidence and analysis. Yrbk. Phys. Anthropol. 50: 106–132.

[74] Ungar PS, Grine FE, Teaford MF (2008) Dental microwear and diet of the Plio-Pleistocene hominin *Paranthropus boisei*. PLoS ONE 3: e2044. Available: doi:10.1371/journal.pone.0002044. Accessed 13 June 2009.

[75] Martins EP (2004) COMPARE, version 4.6b. Computer programs for the statistical analysis of comparative data. Available: http://compare.bio.indiana.edu. Accessed 15 May 2008

[76] Martins EP, Hansen TF (1997) Phylogenies and the comparative method: a general approach to incorporating phylogenetic information into the analysis of interspecific data. Am. Nat. 149: 646–667.

[77] Felsenstein J (1985) Phylogenies and the comparative method. Am. Nat. 125: 1–15.

Figure S1. Phylogeny used in this study. 10kTrees Project (Primates: Consensus tree)

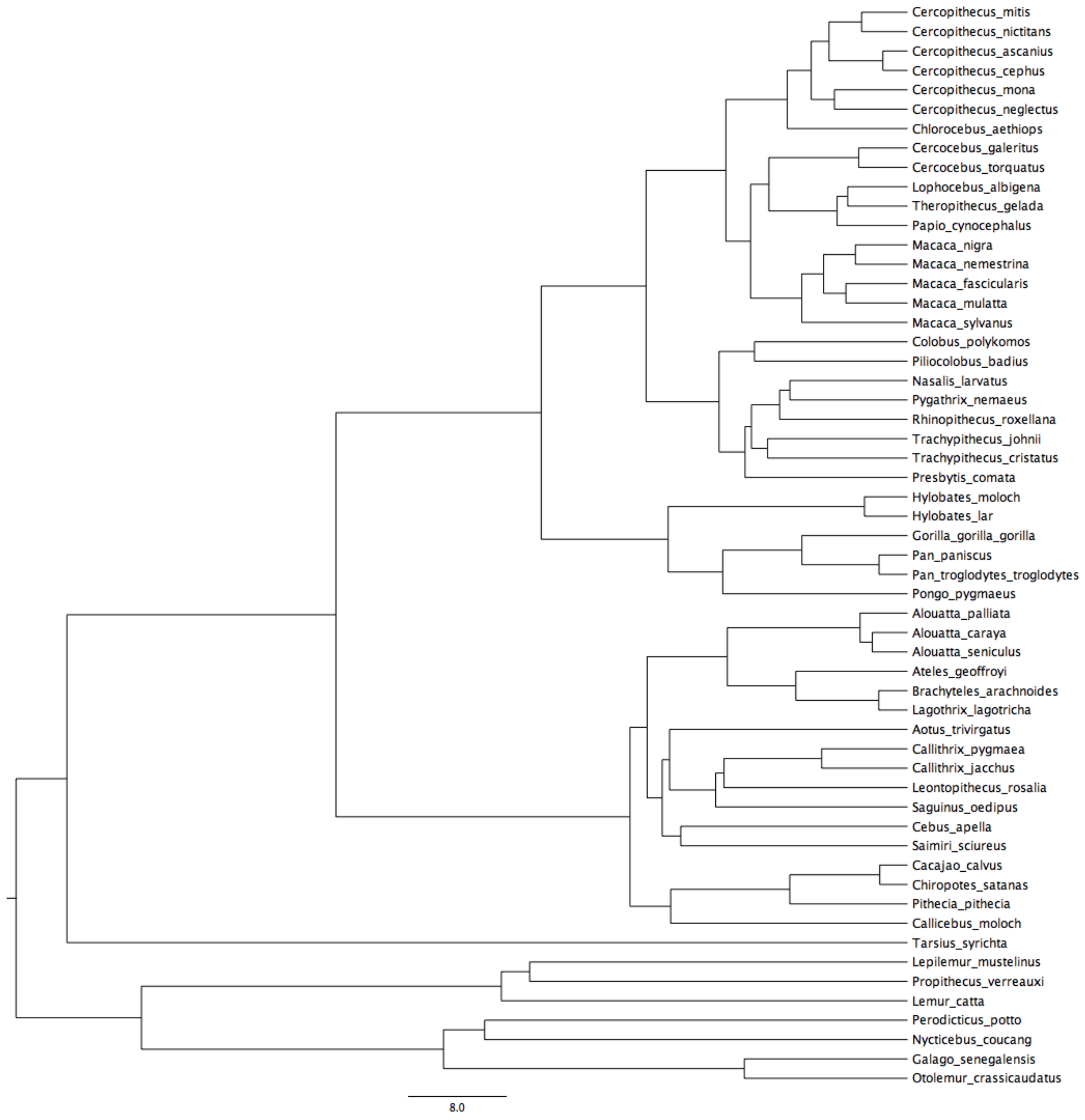

Figure S2. A hypothetical, highly symmetrical phylogeny for eight species from which independent contrasts between taxa can be defined. The tree shows a radiation that gives rise to four pairs of closely related species (modified from Felsenstein [77]).

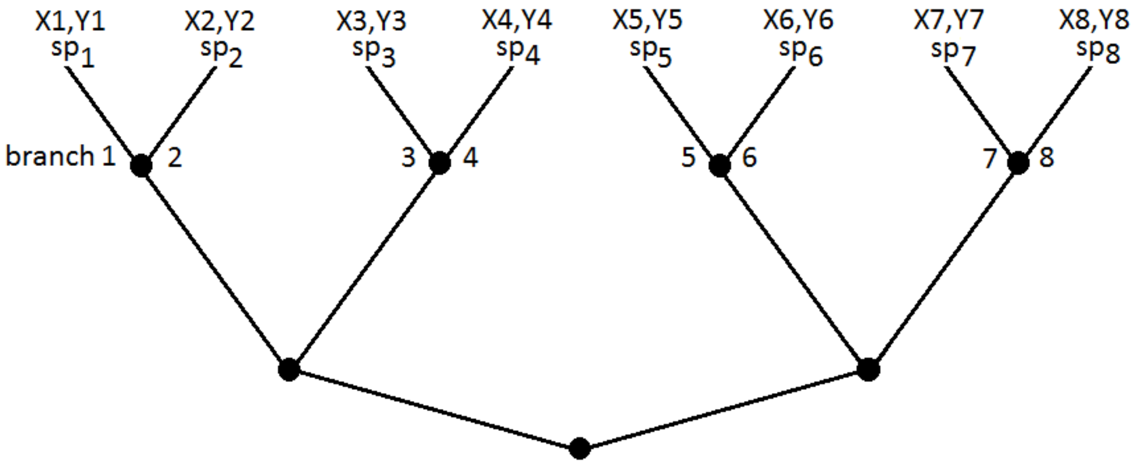

Supplement: Supplementary file 1 — Additional tables and figures, with further discussion of materials and methods as well as additional references not included in the main text. [file 406507.f1.pdf]
